# Hypoxia downregulated miR-4521 suppresses gastric carcinoma progression through regulation of IGF2 and FOXM1

**DOI:** 10.1186/s12943-020-01295-2

**Published:** 2021-01-06

**Authors:** Shan Xing, Zhi Tian, Wenying Zheng, Wenjuan Yang, Nan Du, Yixue Gu, Jiang Yin, Hao Liu, Xiaoting Jia, Donglan Huang, Wanli Liu, Min Deng

**Affiliations:** 1 Affiliated Cancer Hosipital & Institute of Guangzhou Medical University, Guangzhou Key Laboratory of “Translational Medicine on Malignant Tumor Treatment”, No.78, Hengzhigang Road, Guangzhou, 510095 China; 2 Department of Clinical Laboratory, State Key Laboratory of Oncology in South China, Collaborative Innovation Center for Cancer Medicine, Sun Yat-sen University Cancer Center, Guangzhou, 510060 China; 3 Department of Pharmaceutical Sciences, Taneja College of Pharmacy, University of South Florida, Tampa, FL 33612 USA

**Keywords:** miR-4521, Hypoxia, IGF2, FOXM1, Gastric carcinoma, Metastasis

## Abstract

**Background:**

MicroRNAs (miRNAs) show considerable promise as therapeutic agents to improve tumor treatment, as they have been revealed as crucial modulators in tumor progression. However, our understanding of their roles in gastric carcinoma (GC) metastasis is limited. Here, we aimed to identify novel miRNAs involved in GC metastasis and explored their regulatory mechanisms and therapeutic significance in GC.

**Methods:**

The microRNA expression profiles of GC tumors at different stages and at different metastasis statuses were compared respectively using the stomach adenocarcinoma (STAD) miRNASeq dataset in TCGA. Using the above method, miR-4521 was picked out for further study. miR-4521 expression in GC tissues was examined by quantitative reverse transcription polymerase chain reaction (qRT-PCR) and in situ hybridization (ISH). Highly and lowly invasive cell sublines were established using a repetitive transwell assay. Gain-of-function and loss-of-function analyses were performed to investigate the functions of miR-4521 and its upstream and downstream regulatory mechanisms in vitro and in vivo. Moreover, we investigated the therapeutic role of miR-4521 in a mouse xenograft model.

**Results:**

In this study, we found that miR-4521 expression was downregulated in GC tissues compared with adjacent normal tissues and that its downregulation was positively correlated with advanced clinical stage, metastasis status and poor patient prognosis. Functional experiments revealed that miR-4521 inhibited GC cell invasion and metastasis in vitro and in vivo. Further studies showed that hypoxia repressed miR-4521 expression via inducing ETS1 and miR-4521 mitigated hypoxia-mediated metastasis, while miR-4521 inactivated the AKT/GSK3β/Snai1 pathway by targeting IGF2 and FOXM1, thereby inhibiting the epithelial-mesenchymal transition (EMT) process and metastasis. In addition, we demonstrated that therapeutic delivery of synthetic miR-4521 suppressed gastric carcinoma progression in vivo.

**Conclusions:**

Our results suggest an important role for miR-4521 in regulating GC metastasis and hypoxic response of tumor cells as well as the therapeutic significance of this miRNA in GC.

## Introduction

Gastric carcinoma is one of the most common malignancies and imposes a significant burden on global health care. It is estimated that there are 1,033,701 new cases of gastric carcinoma annually, and gastric carcinoma is the third leading cause of cancer death worldwide in 2018 (782,685 deaths, 8.2% of the total), according to the global cancer statistics of 2018 from the International Agency for Research on Cancer/World Health Organization (https://gco.iarc.fr/). Due to the lack of early symptoms, the majority of patients with gastric carcinoma are diagnosed at an advanced stage with metastases in lymph nodes, distant organs, or both. Moreover, to date, there are no effective methods for the treatment of GC patients with metastatic disease [1]. New therapeutic options will become available only if we enhance our understanding of the mechanisms underlying metastatic spread.

MicroRNAs are a class of endogenous small noncoding RNAs with 19–25 nucleotides that regulate gene expression at the posttranscriptional level [2, 3]. These RNAs suppress target mRNA expression, mostly through interaction with the 3′-UTR and regulate a diverse array of cellular activities [4]. Accumulating evidence has demonstrated that dysregulated miRNAs play important roles across various types of human cancers including GC, acting as either oncogenes or tumor suppressors [5, 6]. Insights into the roles of miRNAs in cancers have made them attractive tools and targets for novel therapeutic approaches [7, 8]. miRNAs can regulate a broad set of genes in multiple pathways efficiently and simultaneously, which may reduce the emergence of resistant clones in diseases such as cancer since many simultaneous mutations [9] . Moreover, they can be easily delivered to the target cells due to the small size [10]. Thus, miRNA-based therapies have certain advantages. miRNA-based therapies have two different approaches: restoring the expression of tumor suppressor miRNAs using miRNA mimics, like double-stranded synthetic miRNAs, and inhibition of oncogenic miRNAs by miRNA antagonists, such as antisense oligonucleotides and antagomirs. Delivery of endogenous tumor suppressor miRNAs as synthetic miRNA mimics by a variety of carriers has emerged as a promising approach to treat cancer [11, 12]. For instance, miR-655-3p delivery by nanoscale coordination polymers limits epithelial-mesenchymal transition and suppresses liver metastases of colorectal cancer in mice [13]. Singh et al. reported that miR-215-5p mimic treatment suppresses mesothelioma progression by activating p53 function and inducing apoptosis [14]. Likewise, systemic delivery of miR-34a mimics demonstrated promising results in mouse models of lung [15, 16] and prostate [17] cancer by inhibiting tumor growth and metastasis, with no evidence of adverse effects caused by carrier-mediated immune stimulation. In a recent clinical trial (NCT02369198 and ACTRN12614001248651), a miR-16-based mimic drug exerted antitumor activity, and patients with malignant pleural mesothelioma had an encouraging response and survival [18].

Albeit a large number of preclinical studies on miRNA therapeutics have been conducted over the years, only a small number of miRNA therapeutics have so far moved into clinical development. One of the biggest challenges in developing miRNA-based therapeutics is to identify the best miRNA candidates or miRNA targets for each disease type. Although several GC-implicated miRNAs have been identified [6, 19], the functions and mechanisms of miRNAs in GC progression and metastasis are still poorly understood. Thus, identification of novel miRNA candidates involved in this disease and understanding of their targets and downstream pathways are needed to develop effective miRNA-based therapeutics for gastric carcinoma.

Here, in an attempt to identify the miRNAs that regulate GC progression and metastasis, we discovered the under-expression of miR-4521 in advanced GC and its key role in inhibiting GC metastasis through the direct regulation of IGF2 and FOXM1, and the inactivation of AKT/GSK3β/Snai1 signaling. Hypoxia in the tumor microenvironment contributed to miR-4521 downregulation in an ETS1-dependent manner and miR-4521 mitigated hypoxia-mediated metastasis. Moreover, therapeutic delivery of synthetic miR-4521 suppressed gastric carcinoma progression in vivo, indicating a novel potential target for GC treatment.

## Materials and methods

### Bioinformatics analysis

miRNASeq data from the TCGA stomach adenocarcinoma dataset were used for differential analysis of the microRNA expression profiling of GC tumors at different metastasis statuses and at different stages. According to AJCC TNM staging, Stage I (58 samples) and Stage II (128 samples) GCs were combined to form the early-stage GC cohort, which was compared with the late-stage cohort (Stage III, 180 samples and Stage IV, 43 samples). Metastatic GCs (M1, 30 samples) were compared to non-metastatic GCs (M0, 384 samples).

RNAseq data were obtained from the TCGA stomach adenocarcinoma dataset. GC samples were divided into a miR-4521-high group and a miR-4521-low group according to miR-4521 expression and GSEA was performed on various functional and/or characteristic gene signatures by comparing gene sets from the MSigDB database v3.0.

### Human tissue specimens

Three independent cohorts of human GC samples were used in this study. The frozen samples containing 140 GC tissues and 62 adjacent nontumor tissues (cohort A) used for qRT-PCR assay were gathered from the Affiliated Tumor Hospital of Guangzhou Medical University (Guangzhou, China) from March 2015 to December 2018. Additionally, two GC tissue microarrays used for ISH analysis were enrolled for this study: One containing 90 pairs of GC tissues and adjacent normal samples (cohort B) was obtained from OUTDO Biotech (Shanghai, China); Another including 94 GC tissues and 76 adjacent tissues (cohort C) was provided by the Affiliated Tumor Hospital of Guangzhou Medical University. All patients provided informed consent. This study was approved by the Institutional Review Board of Guangzhou Medical University. The clinical and histopathological characteristics of the patient samples are described in Table S1–3.

### qRT-PCR

Total RNA was isolated from patient tissues and cultured cells using TRIzol reagent (Invitrogen, Carlsbad, CA, USA) according to the manufacturer’s instructions. For mRNA detection, gene expression was analyzed by the SYBR Green qRT-PCR kit according to the manufacturer’s instructions (Takara, Ohtsu, Japan), and the results were normalized to β-actin expression. The primer sequences for each gene are provided in Table S4.

For miRNA detection, cDNA was synthesized with the Mir-X™ miRNA First Strand Synthesis Kit (Takara), and subsequent qPCR analysis was performed using the SYBR Premix Ex Taq Kit (Applied Biosystems, Foster City, CA, USA). U6 snRNA was used as the endogenous control to normalize miRNA expression.

### In situ hybridization

In situ hybridization (ISH) analysis was performed according to previously described methods [20]. Tissue microarray slides were deparaffinized, digested with proteinase K and hybridized with DIG-labeled LNA probes (Exiqon) for miR-4521 and U6 (positive control) at 52 °C overnight and subsequently visualized with an anti-DIG-POD antibody and DAB complex. miR-4521 expression levels were quantified according to the staining intensity and positive percentage of miR-4521. The intensity of staining was scored from 0 to 4, and positive percentage of staining was scored from 0 to 100%. The final quantitation of each staining was obtained by multiplying the two scores.

### Cell culture and hypoxic conditions

The gastric carcinoma cell lines BGC823, SGC7901 and MGC803 were obtained from the Cell Bank of the Chinese Academy of Sciences (Shanghai, China). The breast cancer cell line MCF7 and lung cancer cell line A549 as well as the human embryonic kidney (HEK) 293 T cell line were obtained from the American Type Culture Collection (Manassas VA, USA). Cell lines involved in our experiments were reauthenticated by short tandem repeat analysis every 6 months after resuscitation in our laboratory. These cells were cultured in Dulbecco’s Modified Eagle’s Medium (DMEM, Gibco, USA) supplemented with 10% fetal bovine serum (HyClone, Logan, UT, USA) at 37 °C in a humidified incubator with 95% air and 5% CO_2_. For hypoxic exposure, cells were cultured under 1% oxygen tension (1% O_2_) in a hypoxia chamber.

### Establishment of highly and lowly invasive cell sublines

Highly and lowly invasive cell sublines were established from the SGC7901 and BGC823 cell lines by the method (Fig. S1) described in a previous study [21]. Transwell inserts (Corning Costar, Cambridge, MA, USA) pre-coated with Matrigel on the 8.0 μM permeable polycarbonate membrane were used to isolate cell sublines with different levels of invasiveness from the cultured SGC7901 and BGC823 cell lines. Briefly, 2 × 10^5^ cells in serum-free medium were seeded into the upper chamber, and 500 μl of medium containing 15% fetal bovine serum was added to the lower chamber to create a chemotactic gradient.. After 48 h of incubation at 37 °C, the cells on the upper surface of the membrane and the invading cells on the lower surface were harvested aseptically and expanded for further selection with Matrigel-coated transwell inserts. The cell sublines with highly or lowly invasive abilities were established via ten-round selection and named SGC7901-H, BGC823-H, SGC7901-L and BGC823-L, respectively.

### Vector construction

The 3′-UTR of either IGF2 or FOXM1 was cloned into the dual-luciferase reporter vector pmirGLO (Promega, Madison, WI, USA), termed Luc-IGF2 and Luc-FOXM1, respectively, and subsequently the mutant vectors with point mutations in miR-4521 binding sites were synthesized using the QuikChange Site-Directed Mutagenesis Kit (Stratagene, La Jolla, CA, USA). The ORF cDNA of FOXM1 was cloned into the expression vector pcDNA3.1 and named pFOXM1. To construct the reporter for miR-4521 promoter activity, the miR-4521 promoter sequence (2000 bp of sequence upstream of the transcription start site) was amplified by PCR from genomic DNA and inserted into the vector pGL3 basic (Promega) upstream of the firefly luciferase gene. The predicted ETS1-binding sites in the miR-4521 promoter sequence were further mutated with the QuikChange Site-directed Mutagenesis Kit (Stratagene).

Lentiviral shRNA vectors targeting either HIF1α or FOXM1 were ordered from GeneChem (Shanghai, China), and lentivirus particles were generated by cotransfecting the shRNA vectors and packaging plasmids into HEK293T packaging cells. Recombinant lentiviruses for miR-4521 overexpression or knockdown and ETS1 siRNAs were purchased from RiboBio (Guangzhou, Guangdong, China). ETS1 shRNA lentiviruses were obtained from GeneChem (Shanghai, China).

### Establishment of stable cell lines

Recombinant miR-4521 lentiviruses were transduced into the highly invasive cell lines SGC7901-H and BGC823-H, while lentiviruses for miR-4521 knockdown were infected into the lowly invasive cell lines SGC7901-L and BGC823-L, following the manufacturer’s instructions. The transduced cells were then selected with 2 mg/L puromycin (Invivogen, San Diego, CA, USA) for 2 weeks to obtain cells with stable overexpression or knockdown of miR-4521. To establish the cell line that stably deplete ETS1, SGC7901-H cells were infected with ETS1 shRNA lentiviruses. The infected cells were then selected with 2 mg/L puromycin for 2 weeks.

### Cell invasion and migration assays

For the cell invasion assay, the starved cells suspended in serum-free DMEM were seeded into the upper chamber with Matrigel in the insert of a 24-well culture plate (Corning Costar). Medium containing 15% fetal bovine serum was added to the lower compartment as a chemoattractant. After incubation for 48 h, invasive cells adhering to the lower membrane of the inserts were fixed, stained, counted and imaged. The cell migration ability was measured by the wound healing assay. Cells were placed into 6-well plates and cultured until 90% confluence. An artificial scratch was created using a 10-μL pipette tip, and cells were cultured in serum-free medium. At 0 and 38 h, wound closure images were captured in the same field under magnification. Cell healing rates were calculated by the fraction of cell coverage across the line.

### Cell proliferation assay

Cells were seeded into 6-well plates, and the cell numbers were counted after 1 day, 2 days, 3 days, 4 days, and 5 days of culturing in DMEM supplemented with 10% fetal bovine serum using a Coulter Counter (Beckman Coulter) in triplicate.

### Animal experiments

All animal studies were approved by the Institutional Animal Care and Use Committee (IACUC) of Guangzhou Medical University, and all animals were ethically and humanely treated. BALB/c nude mice were purchased from the Experimental Animal Center of Guangdong (Foshan, Guangdong, China). To investigate the effect of miR-4521 on tumor metastasis in vivo, miR-4521-overexpressing SGC7901-H cells, miR-4521-silenced SGC7901-L cells and their matched control cells were injected into the abdominal cavity or tail vein of BALB/c nude mice to generate peritoneal dissemination or pulmonary metastases, respectively. To determine the role of ETS1 in GC progression in vivo, ETS1-silenced SGC7901-H cells and the control cells were intraperitoneally injected into BALB/c nude mice. The luciferase signal intensity was monitored in vivo using an In Vivo Imaging System (FX PRO, Bruker, Billerica, MA, USA). Then, mice were sacrificed, and metastatic foci in the abdominal cavity and lung were evaluated.

To investigate the therapeutic role of miR-4521 in vivo, SGC7901 cells expressing luciferase were intraperitoneally injected into 8-week-old female nude mice. After 7 days, mice were randomly divided into two groups (5 mice/group) and intraperitoneally injected with 20 mg/kg miR-4521 agomir or control agomir twice per week for 3 weeks. Thirty days after inoculation, the luciferase signal intensity was monitored in vivo by bioluminescence imaging (BLI), and then mice were sacrificed, and intestine, liver and spleen metastatic foci were evaluated. For subcutaneous injection, 1 × 10^6^ SGC7901 cells were injected into the lower back region of nude mice. After approximately 14 days, when tumors reached an average of 100 mm^3^, 200 μg of miR-4521 agomir or control agomir was injected intratumorally every 2 days for 2 weeks. Tumor growth was monitored using the formula V = LW^2^/2.

### Western blot analysis

Western blot analysis was performed using standard procedures. The following primary antibodies were used in the experiments: anti-FOXM1 antibody (Santa Cruz), anti-pAKT antibody (Peprotech, USA), anti-AKT antibody (Cell Signaling Technology, Beverly, MA, USA), anti-pGSK3β antibody (Proteintech) at 1:1000, anti-Snai1 antibody (Cell Signaling Technology), anti-pIGF1R antibody (Abcam, Cambridge, UK), anti-IGF1R antibody (Abcam, Cambridge, UK), anti-E-cadherin antibody (BD Biosciences, USA), anti-Vimentin antibody (Cell Signaling Technology), anti-N-cadherin antibody (Cell Signaling Technology), anti-pERK1/2 antibody (Abcam), anti-ERK1/2 antibody (Abcam), anti-HIF1α antibody (Novus Biologicals, USA), anti-ETS1 antibody (Abcam) and anti-β-actin antibody (Sigma, St. Louis, MO,USA).

### Chromatin immunoprecipitation

The chromatin immunoprecipitation (ChIP) assay was performed using a Chromatin Immunoprecipitation Assay Kit (Millipore, Bedford, MA, USA). SGC7901-L and BGC823-L cells were exposed to hypoxia or normoxia for 36 h, then crosslinked, lysed and sonicated. Immunoprecipitation was performed using anti-ETS1 antibody, anti-GAPDH antibody or IgG. The precipitated DNA was subjected to real-time PCR.

### Luciferase reporter assay

The reporter vector Luc-IGF2, Luc-FOXM1 or the respective mutation vector with miR-4521-binding site mutations was cotransfected with miR-4521 mimic or inhibitor (RiboBio) into HEK-293 T cells. Firefly and Renilla luciferase activities were measured 48 h after transfection using the Dual-Luciferase Reporter Assay System (Promega) according to the manufacturer’s instructions. In addition, the miR-4521 promoter reporter construct with wild-type or mutated ETS1 binding sites was transfected into HEK-293 T cells, and the pTK-Cluc vector was used as the control. These cells were exposed to hypoxia or normoxia for 48 h, and firefly and Renilla luciferase activities were measured using the dual luciferase system.

### Immunohistochemistry

Immunohistochemistry (IHC) staining of mouse tumor sections were performed according to standard protocols. In brief, paraffin-embedded sections were deparaffinized, rehydrated and subjected to antigen retrieval. After blocking with goat serum, sections were incubated with anti-E-cadherin (BD Biosciences) or anti-Vimentin antibody (Cell Signaling Technology) overnight at 4 °C, followed by secondary antibody incubation for 1 h in room temperature. DAB staining was performed with IHC assay kit (Maixin, Fuzhou, China).

### Statistics

All statistical analyses were carried out using SPSS 23.0 software (SPSS, Chicago, IL, USA) or GraphPad PrismV7 (GraphPad Prism, La Jolla, CA, USA). Two groups were compared using Student’s t-test, and three or more groups were analyzed with ANOVA. The Kaplan-Meier method and log-rank test were employed to generate the survival curve and compare differences between survival curves. Cox regression was utilized to estimate the hazard ratio and 95% confidence intervals for survival. Pairwise expression correlation was analyzed by Pearson correlation tests. *P* < 0.05 was considered statistically significant.

## Results

### Downregulation of miR-4521 is associated with disease progression and prognosis in gastric carcinoma

**Fig. 1:**
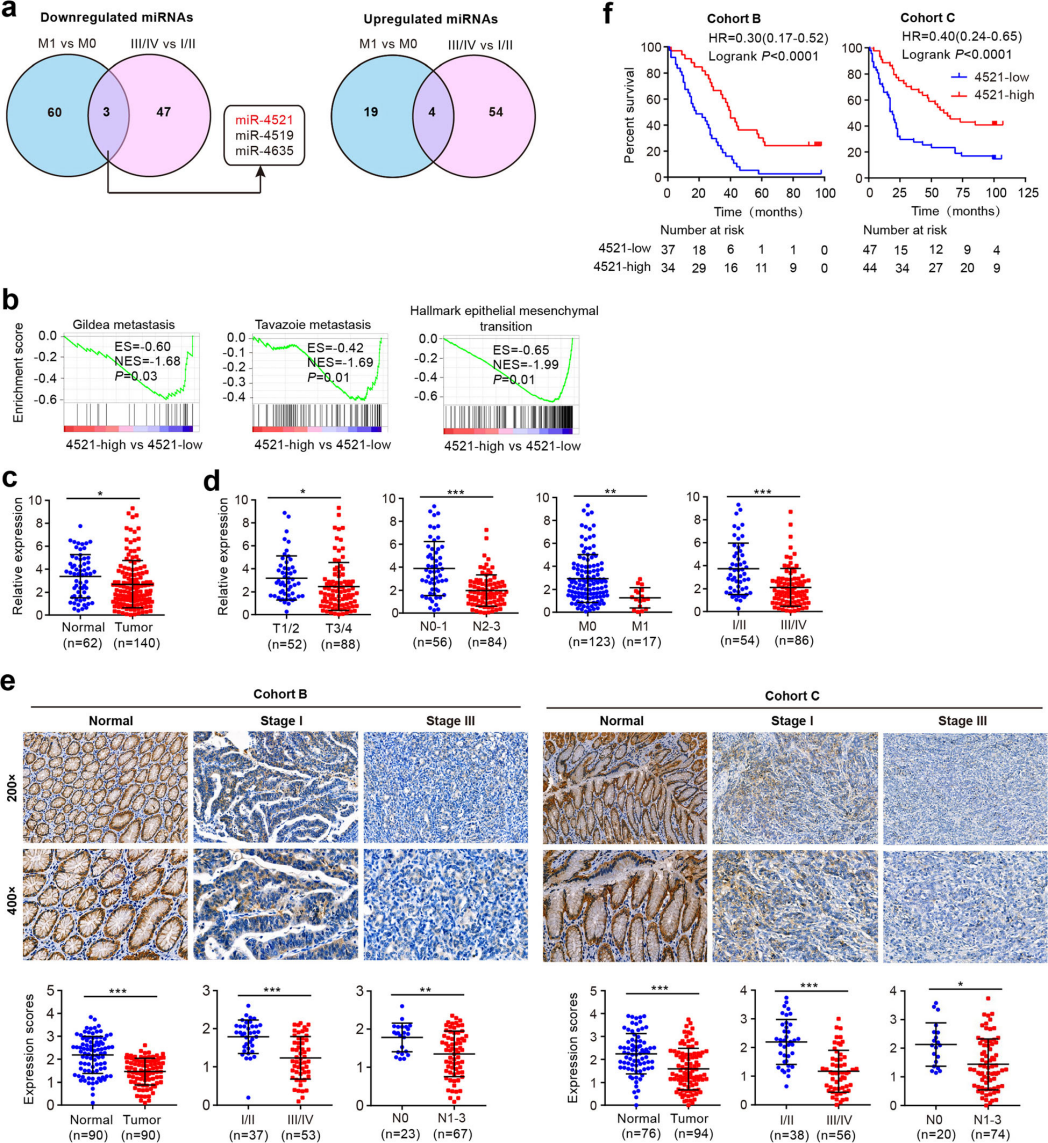
miR-4521 downregulation is associated with disease progression and prognosis in gastric carcinoma. **a** Venn diagram of the differentially expressed miRNAs that are significantly downregulated or upregulated in metastatic vs non-metastatic GCs and late- versus early-stage GCs from stomach adenocarcinoma miRNA-seq data in TCGA. **b** Signatures involved in metastasis and EMT are enriched in miR-4521-high versus miR-4521-low patients by GSEA. ES, enrichment score; NES, normalized enrichment score. **c** qRT-PCR analysis of miR-4521 expression in 140 GC cancer tissues and 62 adjacent normal tissues (cohort A). **d** Assessment of miR-4521 expression levels in GCs according to their clinical stage, T stage, and status of lymph node or distant metastasis. **e** ISH analysis of miR-4521 expression in GC specimens and normal tissues (cohort B and cohort C) on tissue microarrays. **f** Kaplan-Meier analysis of overall survival for patients with GC based on miR-4521 expression. The defined “high” and “low” expression levels of miR-4521 were stratified by the median expression level. **c-e** Error bars indicate SD. *P < 0.05; **P < 0.01; ***P < 0.001

To identify miRNA candidates involved in GC progression and metastasis, we systematically compared microRNA expression levels in metastatic vs nonmetastatic GCs and in late- versus early-stage GCs using the TCGA stomach adenocarcinoma miRNASeq dataset (http://cancergenome.nih.gov/). Differential expression analysis identified 63 miRNAs downregulated and 23 miRNAs upregulated in metastatic vs non-metastatic GCs, while 50 miRNAs downregulated and 58 miRNAs upregulated in late- vs early-stage GC tumors (filtered by *P* < 0.05, Fig. 1a). Of these, miR-4521, miR-4519 and miR-4635 were commonly downregulated miRNAs. At the same time, based on miR-4521 levels, we performed gene set enrichment analysis (GSEA) in a TCGA RNA-seq dataset. Gene sets associated with metastasis and EMT were observed in GC patients with low miR-4521 expression (Fig. 1b), implying a role of this miRNA in GC metastasis.

To confirm the miR-4521 expression levels in GC, we performed qRT-PCR on 140 GC samples and 62 adjacent non-tumor tissues (cohort A) and found that the expression of miR-4521 was significantly downregulated in GC tissues (Fig. 1c). Meanwhile, there was an obvious decrease in miR-4521 expression in metastatic vs non-metastatic GCs, in N2–3 vs N0–1 GCs, in T3/T4 vs T1/T2 GCs and in late- vs early-stage GC tumors (Fig. 1d and Table S5). In another two cohorts of GC specimens (cohort B and cohort C) on TMAs, we further confirmed the downregulation of miR-4521 in GC tissues relative to adjacent normal tissues with ISH analysis (Fig. 1e). Moreover, low miR-4521 expression positively correlated with advanced TNM stage, node metastasis and shorter overall survival of patients (Fig. 1f). Univariate and multivariate analyses indicated that miR-4521 expression was an independent prognostic indicator for overall survival in both cohorts of GC patients (Table S6, 7). These data suggest that downregulation of miR-4521 may be involved in GC progression.

### miR-4521 inhibits GC cell invasion and metastasis in vivo and in vitro

**Fig. 2:**
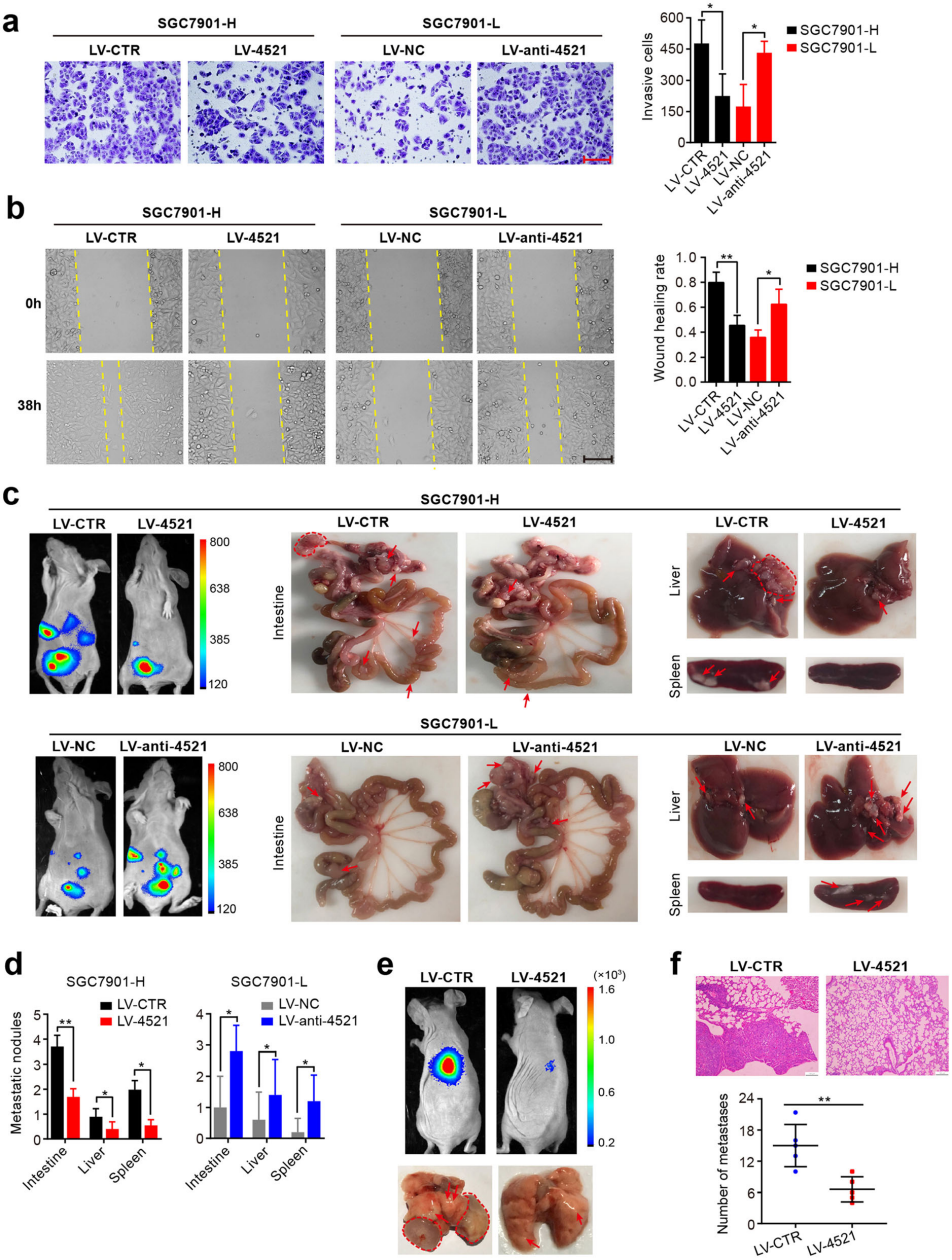
miR-4521 inhibits invasion and metastasis in GC. **a-b** The invasive and migratory abilities of SGC7901-H cells stably expressing miR-4521, SGC7901-L cells stably silencing miR-4521 and the respective control cells were analyzed by transwell and wound healing assays. Scale bars, 150 μm. Error bars, SD from three independent experiments performed in triplicate. *P < 0.05, **P < 0.01. **c-d** Nude mice were intraperitoneally injected with 5 × 10^5^ SGC7901-H and SGC7901-L cells stably expressing the indicated constructs. After 21 days, the metastasis signal was measured by BLI, the mice were sacrificed, and tumor nodules in the abdominal cavity were examined. Representative images of bright view and bioluminescent view are shown. Arrows or circles indicate metastatic nodules. The number of metastatic nodules was quantified. Error bars, SD (*n* = 5 mice/group). *P < 0.05, **P < 0.01. **e-f** SGC7901-H cells with stable miR-4521 overexpression or controls (1 × 10^6^ cells) were injected into the nude mice via the tail vein, with 5 mice per group. Representative images of the luciferase signals of lung metastasis and bright view and H&E staining of lung tissue are shown. The number of metastatic foci was quantified. Scale bars, 100 μm. Error bars indicate SD. **P < 0.01

We subsequently investigated the role of miR-4521 in GC invasion and metastasis. To address this, we first established cell sublines with high invasion ability (SGC7901-H and BGC823-H) and low invasion ability (SGC7901-L and BGC823-L) from the human GC cell lines SGC7901 and BGC823 using the repeated invasion transwell method (Fig. S1). We then detected the miR-4521 expression levels in these cell lines and observed that highly invasive cells had reduced miR-4521 expression compared with that of the parallel low-invasive cells and the individual parental cells (Fig. S2a). Thus, the highly invasive cell lines were selected for the lentiviral-mediated stable overexpression of miR-4521, while the lowly invasive cell lines were used for lentiviral-mediated stable knockdown of miR-4521 (Fig. S2a). We subsequently assessed the metastatic potential of miR-4521. The results from transwell and wound healing assays demonstrated that ectopic expression of miR-4521 in highly invasive cells resulted in a reduction in cell invasion and migration. Conversely, inhibition of miR-4521 in less-invasive cells resulted in an increase in cell invasion and migration (Fig. 2a, b and Fig. S2c, d). In addition, a cell growth curve assay revealed that miR-4521 overexpression markedly reduced cell proliferation, while silencing it had the reverse effect (Fig. S2e).

Subsequently, to observe the effect of miR-4521 on tumor metastasis in vivo, stable miR-4521-expressing SGC7901-H cells and miR-4521-silenced SGC7901-L cells were intraperitoneally or intravenously injected into BALB/c nude mice to generate peritoneal dissemination or pulmonary metastases, respectively. The peritoneal dissemination assays showed that lower metastasis signals were found in the miR-4521-overexpressing group compared with signals in the control group, while miR-4521 suppression in lowly invasive SGC7901 cells led to the opposite effect (Fig. 2c). We also found that the mice injected with miR-4521-overexpressing cells exhibited fewer and smaller tumor nodules in the intestine, mesentery, liver and spleen compared with that in the control group. In contrast, miR-4521 inhibition increased the number of metastases compared with that in the control group (Fig. 2c, d). In the pulmonary metastasis assays, ectopic miR-4521 expression in SGC7901-H reduced lung metastasis, as evidenced by the lower metastasis signals and fewer metastatic foci compared with those of the control (Fig. 2e, f). Collectively, these results demonstrate that miR-4521 inhibits cancer cell invasion and metastasis.

### Hypoxia represses miR-4521 expression through inducing ETS1

**Fig. 3:**
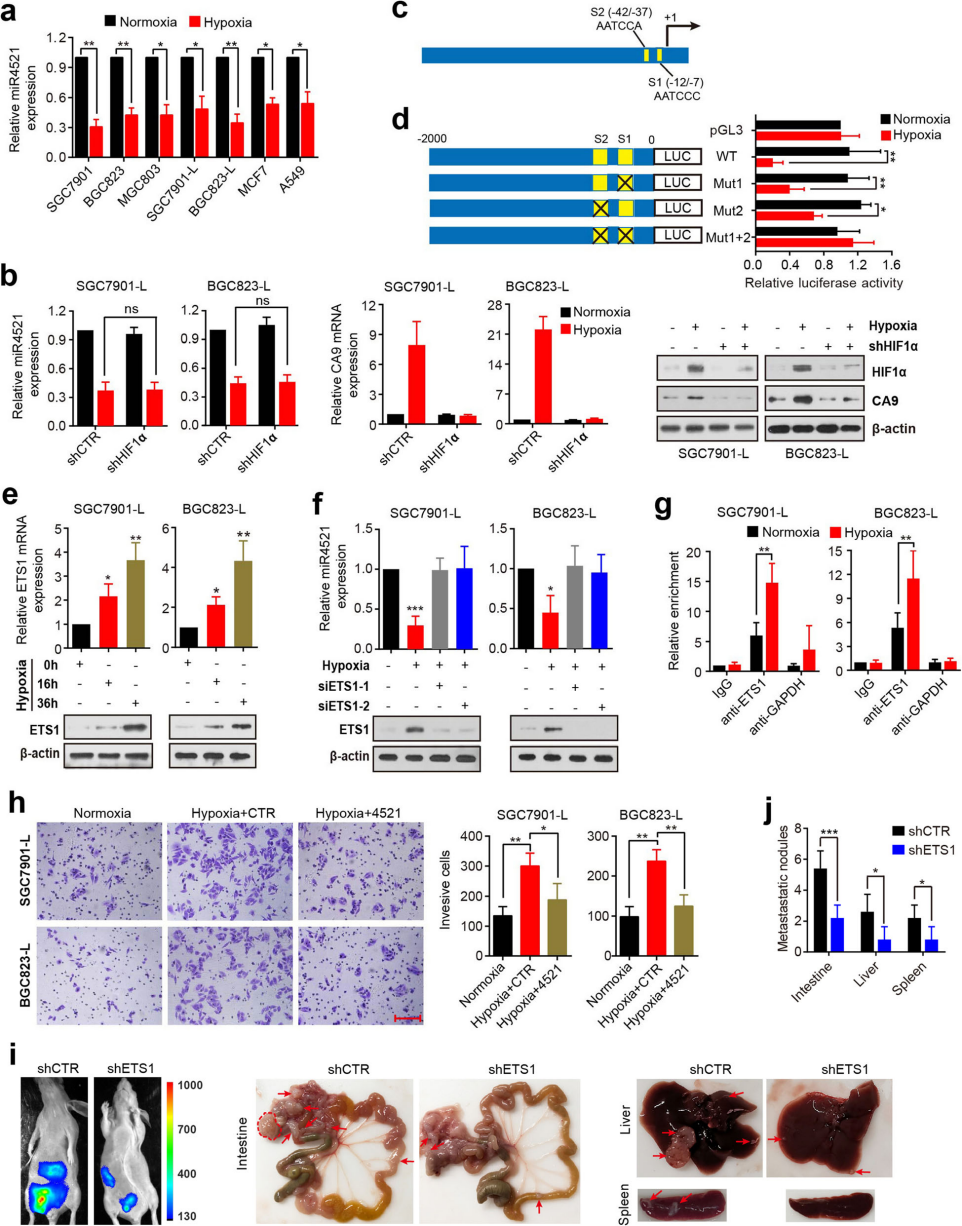
Hypoxia downregulates miR-4521 via ETS1. **a** qRT-PCR analysis of miR-4521 expression under hypoxic conditions (1% oxygen, 36 h) in various tumor cell lines. **b** miR-4521 expression in HIF1α-silenced cells after hypoxia stimulation. CA9 serves as a control for HIF1α defect during hypoxia. Meanwhile, Western blotting was performed to detect HIF1α and CA9 protein levels. **c** A schematic diagram illustrating the two putative ETS1 binding sites (S1 and S2) in the miR-4521 promoter. Bioinformatic analysis of potential ETS1 binding sites in the miR-4521 promoter. **d** Luciferase report assays for the miR-4521 promoter region containing either wild-type (WT) or mutated (Mut1, Mut2, Mut1 + 2) ETS1 binding sites under hypoxic conditions. Hypoxia reduced the luciferase activities in WT cells but not in Mut1, Mut2 and Mut1 + 2 cells. **e** qRT-PCR and Western blot assays of ETS1 expression in SGC7901-L and BGC823-L cells exposed to 1% O_2_ for 16 or 36 h. **f** miR-4521 expression was examined by qRT-PCR analysis in SGC7901-L and BGC-L cells transfected with ETS1 siRNAs during hypoxia (upper). Western blotting was performed to assess the inhibition efficiency in the same cells (lower). **g** ChIP analysis of ETS1 enrichment at the miR-4521 promoter during normoxia or hypoxia. IgG and anti-GAPDH antibodies were used as controls. **h** SGC7901-L and BGC-L cells during hypoxia were infected with miR-4521 lentiviruses and analyzed in cell invasion assays. Scale bar, 150 μm. **i-j** 5 × 10^5^ SGC7901-H cells stably expressing ETS1 shRNA or control shRNA were intraperitoneally into BALB/c nude mice to generate peritoneal dissemination (*n* = 5 mice/group). Representative images of bioluminescent view and bright view are shown. Arrows or circles indicate metastatic nodules. The number of metastatic nodules was quantified. In all cases, error bars indicate SD. *P < 0.05; **P < 0.01; ***P < 0.001; ns, not significant

Having identified the anti-metastatic function of miR-4521, we next investigated the upstream mechanism leading to miR-4521 downregulation. Both experimental and clinical data indicated that hypoxia plays a direct role in driving metastasis [22–24]. Moreover, a recent study demonstrated that hypoxia reduces miR-4521 expression in breast cancer cells [25]. Based on these previous findings, we hypothesized that hypoxia might regulate miR-4521 in GC cells, thus leading to miR-4521 underexpression. To evaluate this hypothesis, we exposed multiple cell lines to hypoxia (1% O_2_) or normoxia and then examined miR-4521 expression in these cell lines. The results showed that miR-4521 expression was suppressed under hypoxic conditions in different GC cell lines (the parental GC cell lines SGC7901, BGC823 and MGC803, and the lowly invasive cell lines SGC7901-L and BGC823-L) and breast carcinoma cell line MCF7 as well as lung carcinoma cell line A549, accompanied by HIF-1α induction (Fig. 3a, b). This hypoxic suppression was consistent across several time points (Fig. S3). Additionally, we treated SGC7901-L and BGC823-L cells with cobalt chloride (CoCl_2_) for Chemically induced hypoxia, and observed that CoCl_2_ treatment resulted in a significant reduction in miR-4521 and a marked induction in HIF-1α in a dose-dependent manner (Fig. S4a). To test the possibility of HIF1α dependency on miR-4521 expression, we knocked down HIF1α with shRNA under normoxic or hypoxic conditions and examined this miRNA expression level. Knockdown of HIF1α failed to affect miR-4521 levels in hypoxia exposed GC cells but decreased the HIF1α target gene carbonic anhydrase-9 (CA9) levels (Fig. 3b), indicating that hypoxia-downregulated miR-4521 was not dependent on HIF1α. Similarly, miR-4521 expression levels in CoCl_2_-treated cells remained unchanged upon HIF1α knockdown (Fig. S4b). We further investigated how hypoxia could regulate miR-4521 expression. Luciferase activity for the reporter constructs containing 2 kb of the miR-4521 promoter showed a significant decrease after exposure to hypoxia (Fig. 3d), suggesting that the mechanism is likely to be transcriptional. We carried out bioinformatics analysis using JASPAR (http://jaspar.genereg.net/) and PROMO (http://alggen.lsi.upc.es/cgi-bin/promo_v3/promo/promoinit.cgi?dirDB=TF_8.3) to identify potential regulatory transcription factors. Bioinformatics analysis revealed that the miR-4521 promoter region had ETS1 binding sites at very close proximity to the transcription initiation site (Fig. 3c). Previous studies have indicated that ETS1 induced by hypoxia reduces the transcription of downstream genes when it binds to regions with very close proximity to the transcription initiation site [26, 27]. Given these previous findings, we evaluated whether the downregulation effect of hypoxia on miR-4521 occurs via ETS1. We observed increased ETS1 expression at both the mRNA and protein levels under hypoxic conditions (Fig. 3e). Importantly, the results from the luciferase activity analysis revealed that the ETS1 binding site mutation on the miR-4521 promoter abolished the effect of hypoxia on promoter luciferase activity (Fig. 3d). We then knocked down ETS1 to study its effect on miR-4521 levels. The results showed that silencing of ETS1 rescued miR-4521 expression in hypoxia-exposed GC cells (Fig. 3f). By chromatin immunoprecipitation (ChIP), we discovered a significant enrichment in the binding of ETS1 to the promoter region of miR-4521 in cells under hypoxic conditions compared with IgG or normoxic controls (Fig. 3g). Taken together, our data indicate that hypoxia downregulates miR-4521 through ETS1. Additionally, ectopic miR-4521 expression reversed hypoxia-mediated cell invasion (Fig. 3h), indicating that hypoxia modulates miR-4521 to promote metastasis.

We next examined whether depletion of ETS1 could affect hypoxia-induced aggressive phenotype. Indeed, depletion of ETS1 by siRNAs led to a significant reduction in invasive ability of hypoxia-exposed cells (Fig. 3f and Fig. S5a). To determine the role of ETS1 in tumor progression in vivo, we used SGC7901-H cells to establish a stable ETS1-silenced cell line by lentiviral-mediated shRNA (Fig. S5b), and then the ETS1-silenced SGC7901-H cells were intraperitoneally injected into BALB/c nude mice, followed by the bioluminescence imaging. When ETS1 was silenced, metastatic nodule formation in the peritoneal cavity was reduced, as evidenced by the diminished luminescence signal and reduced tumor number and size relative to control group (Fig. 3i, j). These results indicate that ETS1 promotes GC invasion and metastasis in vitro and in vivo.

### miR-4521 directly targets IGF2 and FOXM1

**Fig. 4:**
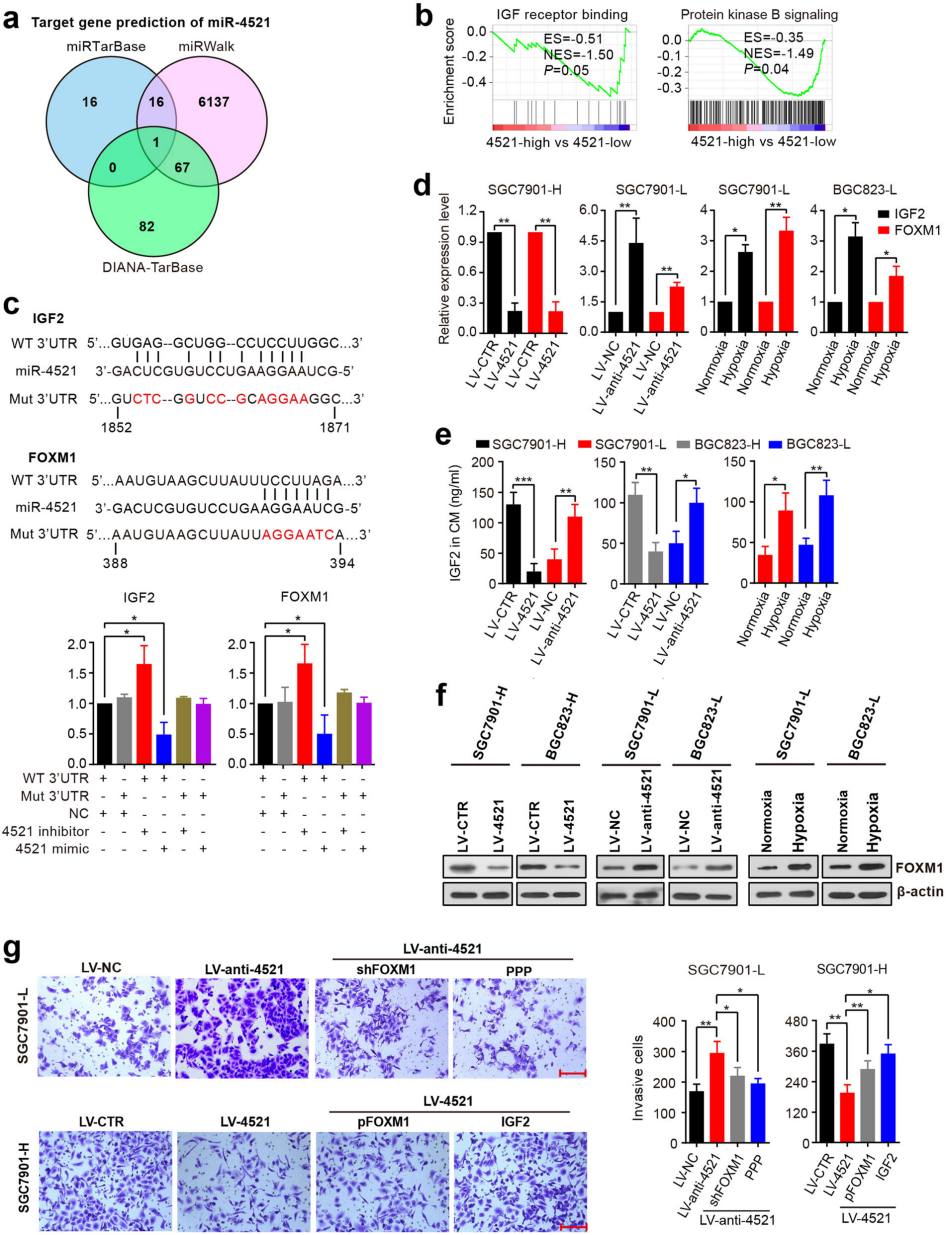
IGF2 and FOXM1 are direct targets of miR-4521 in GC cells. **a** A Venn diagram depicting the overlap of target genes predicted by three miRNA databases (miRTarBase, miRWalk and DIANA-TarBase). **b** GSEA results were plotted to visualize the correlation between the expression of miR-4521 and genes related to the IGF pathway. **c** The 3′-UTRs of IGF2 and FOXM1 had potential miR-4521 binding sites. Luciferase reporter vectors containing WT or mutant IGF2 and FOXM1 3′-UTR were constructed and co-transfected with miR-4521 mimics or inhibitors into HEK293T cells. Luciferase reporter assays were used to determine whether miR-4521 directly binds to the 3′-UTR of FOXM1 or IGF2. **d** RT-qPCR analysis of IGF2 and FOXM1 mRNA expression in SGC7901-H cells with miR-4521 overexpression, SGC7901-L cells with miR-4521 knockdown, and the indicated cells exposed to 1% O_2_ for 36 h. **e** ELISA analysis of IGF2 protein levels in conditioned medium from the indicated cell lines. **f** Western blot analysis of FOXM1 protein levels in SGC7901-H cells stably expressing miR-4521, SGC7901-L cells stably silencing miR-4521 and lowly invasive cells under hypoxic conditions. **g** SGC7901-L cells with miR-4521 knockdown were transfected with FOXM1 shRNA or treated with the IGF1R inhibitor PPP (10 nM) for 48 h, while SGC7901-H cells with miR-4521 overexpression were transfected with a plasmid encoding FOXM1 or treated with 100 ng/ml IGF2 for 48 h. Cell invasion was measured by transwell assay. Scale bar, 150 μm. In all cases, error bars denote SD of triplicates. *P < 0.05; ** *P* < 0.01; ***P < 0.001

**Fig. 5:**
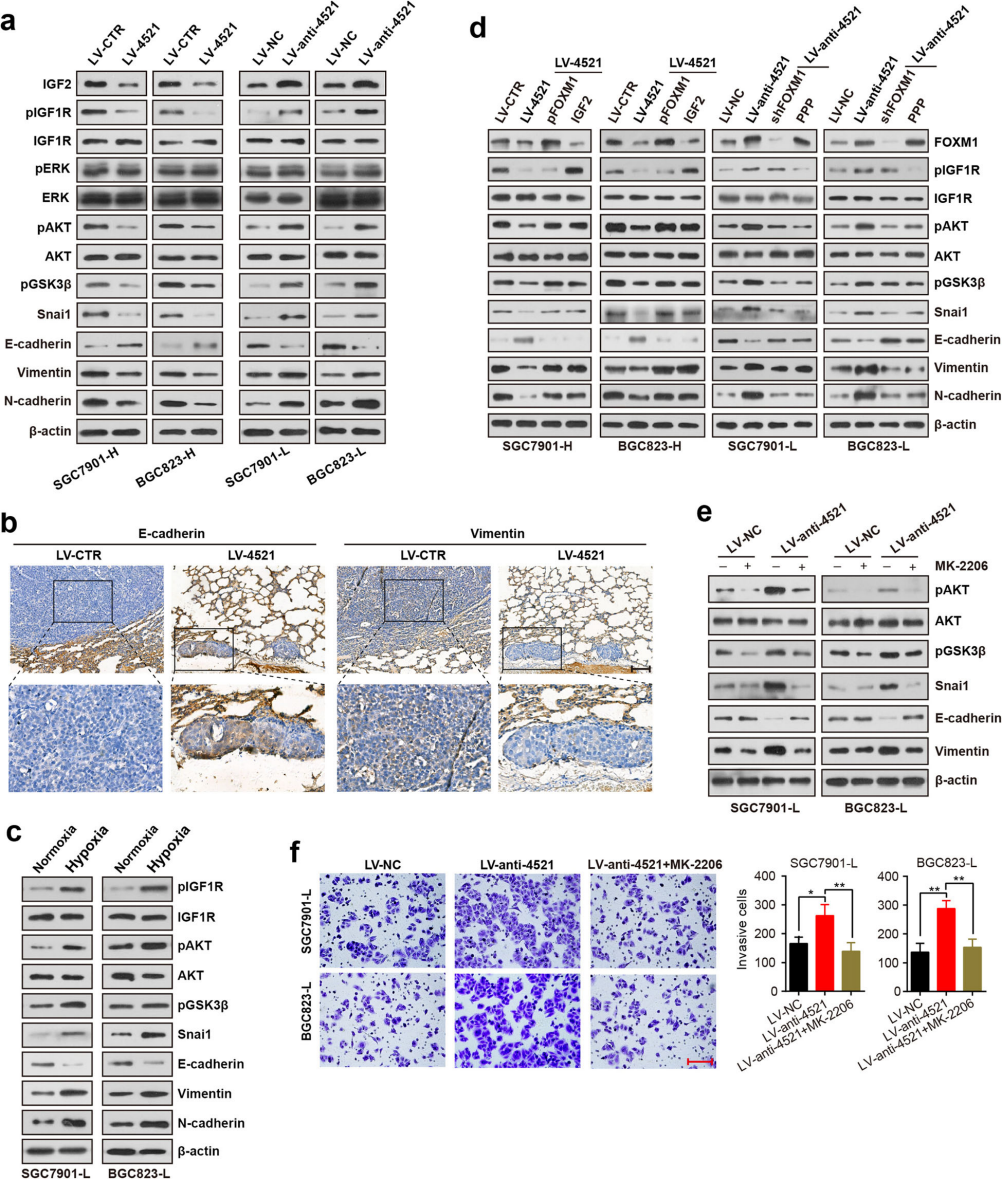
miR-4521 inactivates the AKT/GSK3β/Snai1 pathway. **a** Western blot analysis of the protein expression in miR-4521-overexpressing cells, miR-4521-knockdown cells and control cells. **b** Immunohistochemistry analysis of E-cadherin and Vimentin in mouse lung tumors generated by miR-4521-overexpressing SGC7901-H cells and the control cells. Scale bar, 100 μm. **c** Analysis of the protein expression in the indicated cells in hypoxia or normoxia. **d** miR-4521-expressing cells were transfected with a plasmid encoding FOXM1 or treated with 100 ng/ml IGF2 for 24 h, while miR-4521-silenced cells were transfected with FOXM1 shRNA or treated with PPP (10 nM) for 24 h. Western blotting was performed to detect protein expression. **e** miR-4521-silenced cells were treated with the AKT inhibitor MK2206 (0.5 μM) for 24 h, and the protein expression was measured by Western blotting. **f** Cell invasion ability of miR-4521-silenced cells that were treated with AKT inhibitor MK2206 (0.5 μM) for 48 h. Scale bar, 150 μm. Experiments were repeated three times, and error bars represent SD. *P < 0.05; **P < 0.01

Since miRNAs exert their function by modulating target genes, it is important to identify the target genes of miR-4521 in GC. Using three target gene prediction programs (miRTarBase, miRWalk and DIANA-TarBase), we identified 84 potential targets, such as IGF2 and FOXM1, by at least two programs (Fig. 4a and Table S8). Interestingly, GSEA analysis demonstrated that signature genes involved in IGF signaling were present in the miR-4521-low group (Fig. 4b), implying that miR-4521 regulates the IGF signaling pathway. Given these findings, IGF2 was selected for further verification. An additional candidate, FOXM1, was selected because FOXM1 has been reported to be a target of miR-4521 in medulloblastoma [28]. To test whether they are targets of miR-4521, the 3′-UTRs of the two genes were cloned downstream of firefly luciferase. The luciferase reporters were then co-transfected with miR-4521 mimic or inhibitor into HEK-293 T cells. As shown in Fig. 4c, overexpression of miR-4521 significantly decreased the luciferase activity of the vector containing the IGF2 or FOXM1 3′-UTR, while knockdown of miR-4521 resulted in the opposite effect. Moreover, mutation in the putative miR-4521 seed regions in the 3′-UTRs of either IGF2 or FOXM1 abrogated the suppression of miR-4521. Expectedly, overexpression of miR-4521 decreased IGF2 and FOXM1 mRNA and protein expression levels. Conversely, silencing of miR-4521 elevated IGF2 and FOXM1 levels (Fig. 4d-f, Fig. 5a and Fig. S6). Consistent with this, hypoxia also upregulated IGF2 and FOXM1 expression (Fig. 4d-f). Therefore, miR-4521 targets IGF2 and FOXM1 by directly binding to their 3′-UTRs.

To elucidate whether the metastasis-suppressive effects of miR-4521 were mediated by IGF2 and FOXM1, we treated miR-4521-knockdown cells with either IGF1R (IGF2 receptor) inhibitor PPP or FOXM1 shRNA. In invasion and migration assays, we showed that treatment with IGF1R inhibitor or FOXM1 shRNA abrogated the effect of miR-4521 knockdown on cell invasion and migration. In contrast, recombinant IGF2 treatment or re-expression of FOXM1 rescued miR-4521-inhibited cell invasion and migration (Fig. 4g and Fig. S7, 8). Collectively, these data suggest that miR-4521 suppresses GC metastasis by targeting IGF2 and FOXM1.

### miR-4521 inactivates the AKT/GSK3β/Snai1 pathway

IGF2 is known to bind to type-1 insulin-like growth factor receptor (IGF1R), which activates downstream members of the PI3K/AKT and MAPK/ERK pathways [29, 30]. Therefore, we investigated the possibility that miR-4521 regulates those pathways by targeting IGF2. As expected, miR-4521 overexpression inactivated the IGF pathway, as indicated by the lower phosphorylation levels of IGF1R and AKT and its downstream target GSK3β, whereas opposite results were observed upon miR-4521 knockdown (Fig. 5a). Intriguingly, MAPK/ERK signaling exhibited mild or no changes after either miR-4521 overexpression or knockdown, indicating that miR-4521 specifically inactivates the AKT pathway (Fig. 5a). It is well known that Snai1 is a key downstream factor in the AKT/GSK3β signaling pathway and plays an important role in EMT and metastasis [31, 32]. We subsequently tested this protein level and EMT markers expression with Western blotting and demonstrated decreased expression of Snai1, increased expression of epithelial marker expression (E-cadherin) and reduced expression of mesenchymal markers (Vimentin and N-cadherin) upon miR-4521 overexpression (Fig. 5a). IHC analysis of EMT markers in the xenograft tumor tissues confirmed increased E-cadherin and decreased Vimentin in xenograft tumors derived from miR-4521-overexpressing cells (Fig. 5b). However, miR-4521 knockdown resulted in enhanced Snail expression and EMT phenotype (Fig. 5a). Similarly, the IGF signaling pathway could be activated and EMT was induced after hypoxic stimulation (Fig. 5c). These data suggest the negative regulation of AKT/GSK3β/Snai1 pathway by miR-4521 in GC.

To study whether miR-4521 suppresses the AKT/GSK3β/Snai1 pathway via IGF2, we treated miR-4521-expressing cells with IGF2. The data revealed that the downregulation of IGF1R/AKT/GSK3β/Snai1 signaling by miR-4521 could be rescued upon IGF2 stimulation (Fig. 5d). On the contrary, IGF1R inhibitor treatment effectively reversed the activation effect of miR-4521 knockdown on IGF1R/AKT/GSK3β/Snai1 pathway, suggesting that inactivation of IGF2 interferes with the negative regulation of the AKT/GSK3β/Snai1 pathway by miR-4521 in GC cells. FOXM1 has been reported to activate the AKT/GSK3β/Snai1 pathway [33], although the mechanism responsible for this reactivation remains unclear, and we thus examined whether miR-4521 suppresses the AKT/GSK3β/Snai1 pathway via FOXM1. Indeed, FOXM1 silencing attenuated the activation of the AKT/GSK3β/Snai1 pathway but not the upstream effector IGF1R. On the contrary, the downregulation of AKT/GSK3β/Snai1 by miR-4521 could be rescued by FOXM1 re-expression (Fig. 5d).

To further determine whether miR-4521 could elicit its inhibitory effects by at least suppressing AKT/GSK3β/Snai1 signaling mediator molecules, we used the AKT inhibitor MK2206 to treat miR-4521-knowdown cells. Inhibited activity of the AKT pathway and reduced EMT were observed after MK2206 treatment (Fig. 5e). More importantly, blocking AKT signaling reversed miR-4521 silence-induced invasion and migration (Fig. 5f and Fig. S9). These data indicate that AKT/GSK3β/Snai1 signaling plays an essential function during miR-4521-inhibited GC metastasis.

**Fig. 6:**
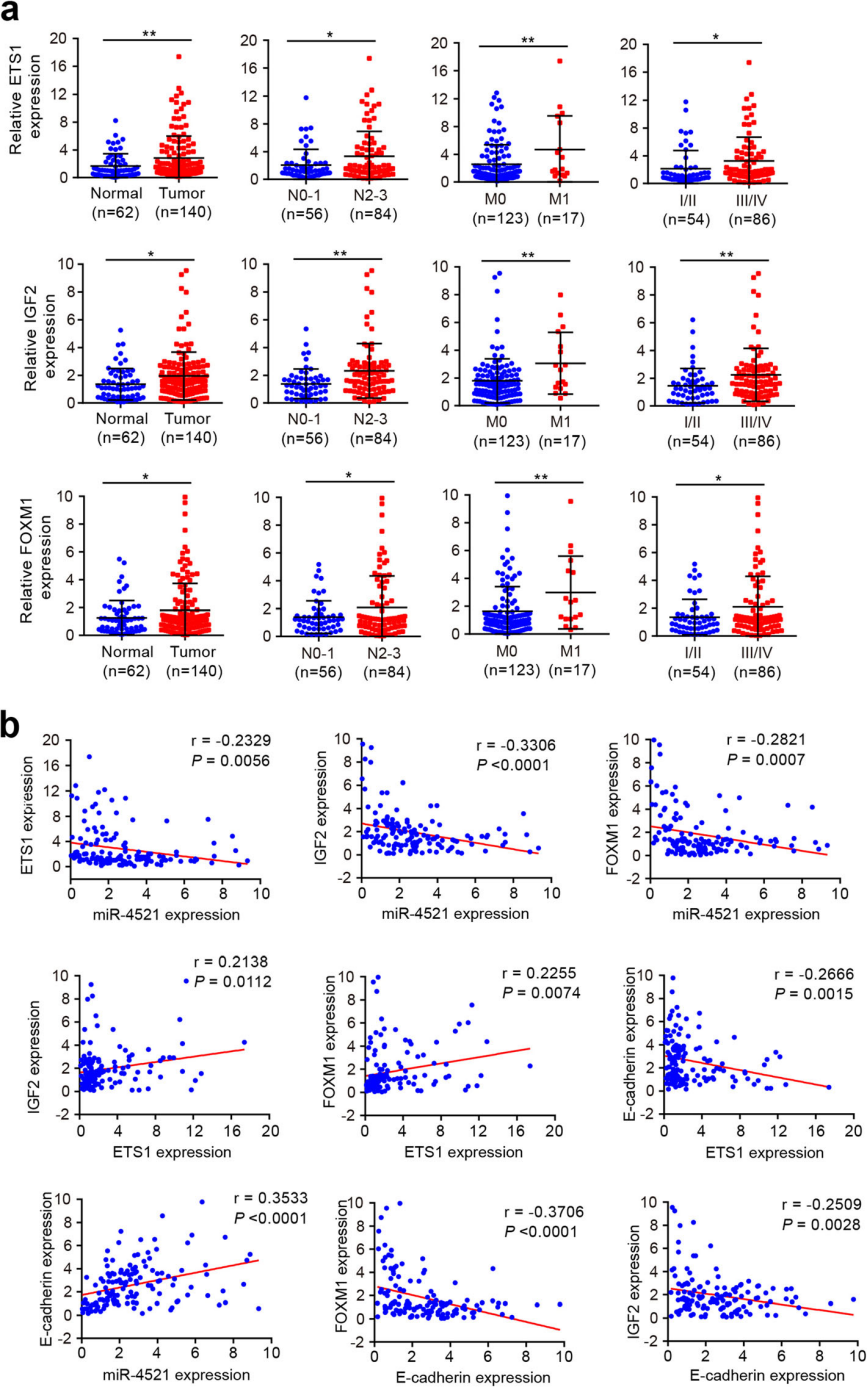
The expression levels of ETS1, IGF2, FOXM1 and E-cadherin and their correlation with miR-4521 expression in GC. **a** Comparison of ETS1, IGF2 and FOXM1 mRNA levels in GC tumors vs normal gastric tissues, N2–3 vs N0–1 GC specimens, metastatic vs non-metastatic GCs and late- vs early-stage GCs, respectively. Error bars denote SD. *P < 0.05; **P < 0.01. **b** miR-4521 expression inversely correlated with ETS1, IGF2 and FOXM1 expression but positively correlated with E-cadherin expression in gastric carcinoma specimens. Moreover, ETS1 expression was positively associated with IGF2 and FOXM1 but negatively associated with E-cadherin in GCs

We then sought to verify whether our findings could be extended to patients with gastric carcinoma. The expression levels of ETS1, IGF2, FOXM1 and E-cadherin were detected by qRT-PCR in the collection of human gastric carcinoma specimens and adjacent normal tissues (cohort A). IGF2, FOXM1 and ETS1 expression was significantly increased in tumors when compared to that in normal tissues. Moreover, GCs with metastasis expressed higher levels of ETS1, IGF2 and FOXM1 than those without metastasis (Fig. 6a). High IGF2, FOXM1 and ETS1 levels also were significantly associated with lymphatic invasion and advanced tumor stage (Fig. 6a). However, E-cadherin displayed the opposite expression model (Fig. S10). Importantly, ETS1, IGF2 and FOXM1 were inversely correlated with miR-4521 and E-cadherin, whereas ETS1 expression was positively associated with IGF2 and FOXM1 expression in GC tissues (Fig. 6b), further confirming the ETS1-miR-4521-IGF2/FOXM1 regulatory axis in GC.

### Therapeutic delivery of miR-4521 suppresses gastric carcinoma progression in vivo

**Fig. 7:**
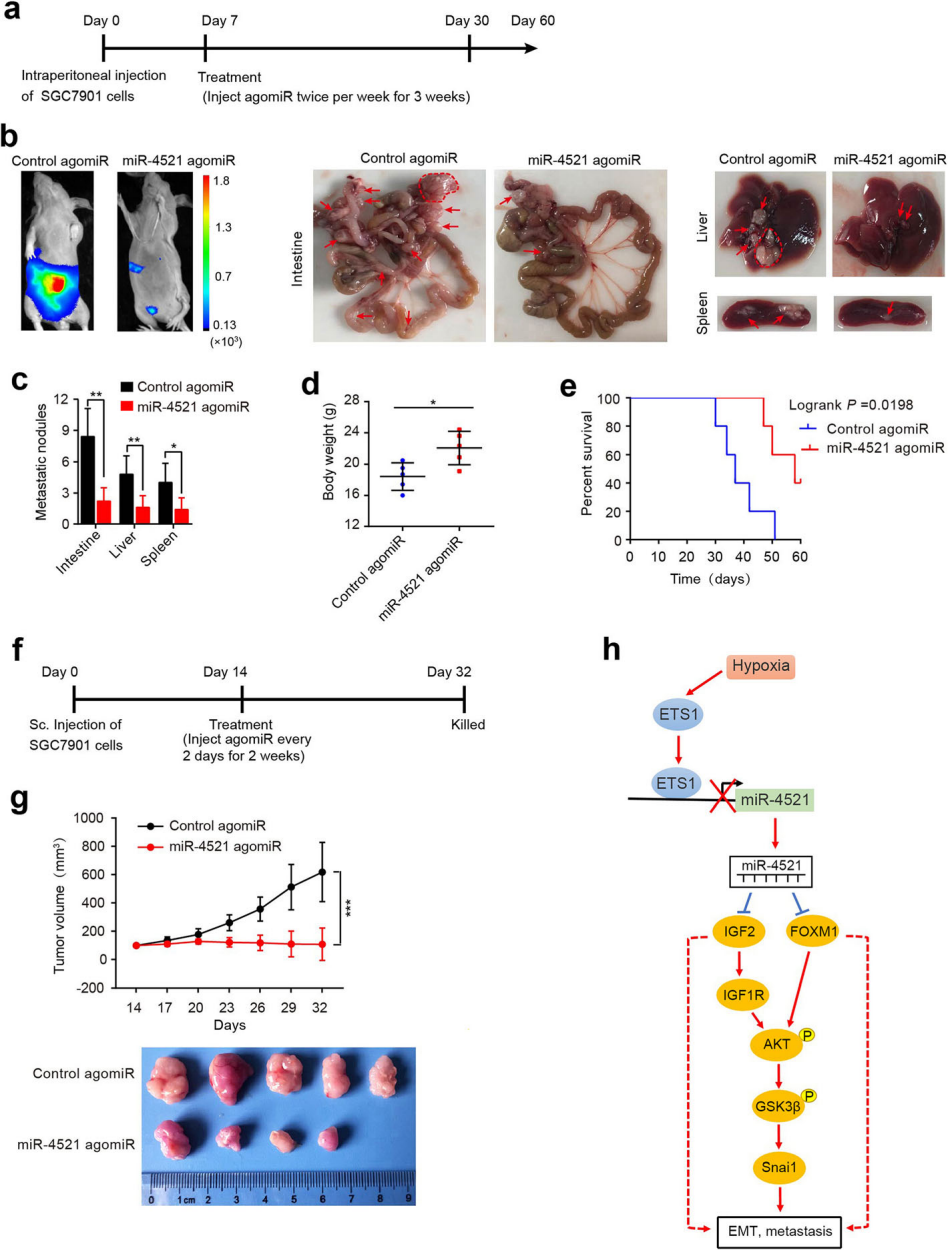
Therapeutic delivery of miR-4521 suppresses gastric carcinoma progression. **a-d** SGC7901 cells expressing a luciferase reporter (1 × 10^6^ cells) were intraperitoneally injected into the nude mice. After 7 days, miR-4521 or control agomiR was delivered by intraperitoneal injection twice per week for 3 weeks (*n* = 5 mice/group) **(a)**. At 30 days after cell injection, bioluminescence imaging and examination of intestinal, hepatic and splenic metastatic nodules showed that miR-4521 agomiR treatment significantly inhibited tumor metastasis **(b, c)**. Arrows or circles indicate metastatic nodules. The body weight of mice is shown **(d)**. Error bars represent SD, *P < 0.05; **P < 0.01. **e** Kaplan-Meier analysis of survival for mice treated with miR-4521 or control agomiR. **f-g** Mice were subcutaneously injected with 1 × 10^6^ SGC7901 cells. Once tumors reached an average of 100 mm^3^ (14 days), the mice were treated with miR-4521 agomiR or control agomiR by intratumoral injection and closely monitored for tumor growth. The mice were sacrificed, and tumors were removed 32 days after inoculation. Error bars, SD (*n =* 5 mice/group). ***P < 0.001. **h** A schematic illustration of Hypoxia/ETS1/miR-4521/IGF2&FOXM1/AKT/Snai1 signaling pathway. Hypoxia downregulates miR-4521 through inducing ETS1. miR-4521 inactivates AKT/GSK3β/Snai1 signaling by suppressing IGF2 and FOXM1

Finally, to investigate the therapeutic role of miR-4521 in vivo, luciferase-expressing SGC7901 cells were intraperitoneally injected into nude mice. After 7 days, miR-4521 or control agomiR was delivered by intraperitoneal injection (Fig. 7a). Luminescence imaging showed that miR-4521 treatment significantly inhibited tumor metastasis compared to that of control treatment (Fig. 7b). Mice treated with miR-4521 agomiR exhibited a dramatically smaller number of macroscopic nodules in the peritoneal cavity (Fig. 7b, c), with higher body weight (Fig. 7d). Furthermore, Kaplan-Meier analysis showed that mice treated with this miR-4521 agomiR exhibited significantly increased overall survival (Fig. 7e).

Additionally, subcutaneous xenografts were established in NOD nude mice using SGC7901 cells. After approximately 14 days, when tumors reached an average of 100 mm^3^, miR-4521 or control mimic agomiR was delivered by intertumoral injection (Fig. 7f). miR-4521 treatment significantly inhibited tumor growth compared to control treatment (Fig. 7g).

## Discussion

Metastasis is one of the most important hallmarks of malignancy and a major cause of cancer-related deaths [34]. To date, there are no specific treatments targeting disseminated disease. Thus, understanding the molecular mechanisms underlying metastasis is essential for identifying novel therapeutic targets and consequently improving the treatment of metastatic diseases, including GC. Emerging evidence has suggested that miRNAs promote or suppress tumor metastasis [5], providing a new perspective on the metastatic process. In this study, we found that miR-4521 was significantly decreased in progressed GC and decreased miR-4521 was associated with an unfavorable prognosis of patients with GC. Functional studies demonstrated that miR-4521 inhibited GC migration, invasion and metastasis.

Very little is known about miR-4521. In 2014, this microRNA was first reported to be downregulated by hypoxia in breast cancer cells [25]. Zhuang linked miR-4521 overexpression to the neural differentiation of human Wharton’s jelly mesenchymal stem cells in 2015 [35]. More recent studies have described decreased expression of miR-4521 in chronic lymphocytic leukemia (CLL) and lung cancer [36] and correlated it to better disease-free survival of patients with esophageal adenocarcinoma [37]. Feng X et al. have also reported that miR-4521 overexpression decreases cell proliferation, migration and invasion of renal cancer cells [38]. To our knowledge, our study is the first to show the potential of miR-4521 in GC, especially GC metastasis.

The hypoxic microenvironment is one of the major features of solid tumors and is strongly associated with poor prognosis in multiple sites, including gastric carcinoma [39]. Clinical studies show a strong association between hypoxia and distant metastasis or relapse [22, 40]. Experimental data have shown that hypoxia prompts tumor metastasis in various tumors [41, 42]. We found that hypoxia in the tumor microenvironment contributes to the repression of miR-4521 expression in a HIF1α-independent manner in GC cell. Previous reports have shown that hypoxia mediates EMT via direct activation of Twist1 by HIF1α [43] or notch signaling [44]. Our findings provide a new understanding of the mechanisms by which hypoxia contributes to EMT and metastasis via ETS1-mediated miR-4521 downregulation.

As part of our research on how miR-4521 suppresses GC metastasis, we showed that IGF2 and FOXM1 were the critical downstream targets of miR-4521. IGF2 is a paternally imprinted growth factor that is highly expressed during embryonic development. The upregulation of IGF2 has been widely observed in many types of human tumors and could be a potential driver in carcinogenesis and tumor metastasis [45]. IGF2 signals through IGF1R, which is one of the crucial receptor tyrosine kinases implicated in tumor development [46]. IGF2 and its receptor IGF1R thus constitute desirable therapeutic targets. Evidence shows that targeting either IGF2 or its receptor IGF1R blocks cancer progression and displays significant antitumor activity [47, 48]. The IGF2 monoclonal antibody Xentuzumab blocking this target is currently undergoing clinical testing in solid tumors [49]. Therefore, the finding that miR-4521 can repress IGF2 signaling provides a rationale for the treatment of gastric carcinoma with miR-4521.

FOXM1 is a member of the forkhead/winged helix box class O (FOXO) superfamily, and this transcription factor plays multiple roles in biological functions, such as facilitating tumor metastasis, cell proliferation, differentiation, invasion, resistance to stress, and DNA damage [50]. Overexpression of FOXM1 has been frequently reported in various types of cancers, including GC [51]. Evidence suggests that FOXM1 promotes GC cell migration, invasion, and EMT and is an independent factor affecting the prognosis of gastric cancer [52, 53]. miR-4521 has been reported to target FOXM1 in medulloblastoma [28]. Here, we confirmed that FOXM1 was a bona fide target of miR-4521 in GC, further supporting the anti-metastatic function of miR-4521.

Previous studies have revealed that activation of the AKT/GSK3β/Snail pathway is required for the induction and maintenance of EMT [54, 55]. In this manuscript, we reported that miR-4521 inactivates the AKT/GSK3β/Snail pathway through IGF2 and FOXM1, two important regulators of AKT signaling, which results in inhibition of EMT and metastasis.

miRNA-based anti-cancer strategies hold great promise for improving outcomes [12], especially in metastatic cancer in which conventional therapies have limited efficacy. In the current study, we confirmed the therapeutic role of miR-4521 in nude mice treated with synthetic agomiR. The use of miR-4521 against GC could be a potentially potent approach because of its resistance against the hypoxia-mediated effects and its mechanism of action targeting IGF signaling and AKT pathway.

Although miR-4521 delivery confers dramatic tumor protection, the systemic and cellular toxicity and immune response caused by either systemic or local delivery of this miRNA requires further investigation. In addition, the efficient delivery of miRNAs to target tissues is so far one of major challenges in the transition of miRNA therapy to the clinic. Thus, the delivery systems that confer higher stability to miR-4521 and enable tissue-specific targeting, as well as avoiding potential toxicities and off-target effects, deserve further investigation. In conclusion, our investigation elucidated that that miR-4521 possesses anti-metastatic activity in GC, and this microRNA functions by targeting IGF2 and FOXM1 and inactivating the AKT/GSK3β/Snai1 pathway (Fig. 7h). Hypoxia in the tumor microenvironment leads to miR-4521 downregulation via inducing ETS1 and this miRNA can reduce hypoxic response of tumor cells. Our finding that synthetic miR-4521 could inhibit GC progression and improve survival provides biological rationale for the use of synthetic miR-4521 mimic as a novel therapeutic strategy for the treatment of gastric carcinoma.

## Supplementary Information

**Additional file 1.**

**Additional file 2.**

## Data Availability

All data and materials can be provided upon request.

## Abbreviations

GC: Gastric carcinoma
miRNAs: microRNAs
EMT: Epithelial-mesenchymal transition
IGF2: Insulin-like growth factor 2
FOXM1: Forkhead box M1
IGF1R: Type-1 insulin-like growth factor receptor
CA9: Carbonic anhydrase-9
qRT-PCR: Quantitative real-time polymerase chain reaction
ISH: In situ hybridization
ChIP: Chromatin immunoprecipitation
TCGA: The Cancer Genome Atlas
GSEA: Gene set enrichment analysis
CLL: Chronic lymphocytic leukemia
DMEM: Dulbecco’s Modified Eagle’s Medium
shRNA: Short hairpin RNA
siRNA: Small interfering RNA
