# Supplementary material for: Hypoxia downregulated miR-4521 suppresses gastric carcinoma progression through regulation of IGF2 and FOXM1
Source: Mol Cancer. 2021 Jan 6;20:9. doi: 10.1186/s12943-020-01295-2 (PMC7786912; doi:10.1186/s12943-020-01295-2)
Supplement: Supplementary file 1 — Additional file 1. [file 12943_2020_1295_MOESM1_ESM.zip › Table S1-7.docx]

**Table S1. Clinicopathological characteristics of cohort A samples.**

| **Viable** | **Cases** |
| --- | --- |
| Median age (range) | 59 years (37-79) |
| Gender  Male  Female | 87 (62.1%)  53 (37.9%) |
| Invasion depth  T1  T2  T3  T4 | 8 (5.7%)  44 (31.4%)  56 (40.0%)  32 (22.9%) |
| TNM stage  I  II  III  IV | 13 (9.3%)  41 (29.3%)  69 (49.3%)  17 (12.1%) |
| Lymph node metastasis  N0  N1  N2  N3 | 12 (8.6%)  44(31.4%)  46 (32.9%)  38 (27.1%) |
| Distant metastasis  M0  M1 | 123 (87.9%)  17 (12.1%) |

**Table S2. Clinicopathological characteristics of cohort B samples.**

| **Viable** | **Cases** |
| --- | --- |
| Median age (range) | 66.5 years (34-83) |
| Gender  Male  Female | 70 (77.8%)  20 (22.2%) |
| Tumor size  <5cm  ≥5cm | 36 (40.0%)  54 (60.0%) |
| TNM stage  I  II  III  IV | 7 (7.8%)  30 (33.3%)  49 (54.4%)  4 (4.4%) |
| Lymph node metastasis  N0  N1  N2  N3 | 23 (25.6%)  16(17.8%)  25 (27.8%)  26 (28.9%) |

**Table S3. Clinicopathological characteristics of cohort C samples.**

| **Viable** | **Cases** |
| --- | --- |
| Median age (range) | 66 years (41-81) |
| Gender  Male  Female | 58 (61.7%)  36 (38.3%) |
| Tumor size  <5cm  ≥5cm | 38 (38.3%)  56 (61.7%) |
| TNM stage  I  II  III  IV | 8 (8.5%)  30 (31.9%)  50 (53.2%)  6 (6.4%) |
| Lymph node metastasis  N0  N1  N2  N3 | 20 (21.3%)  20 (21.3%)  30 (31.9%)  24 (25.5%) |

**Table S4. The primers used for qRT-PCR in this study.**

| **Primer** | [**Sequence**](D:/program%20files/Youdao/Dict/8.5.1.0/resultui/html/index.html#/javascript:;)**(5′→3′)** |
| --- | --- |
| IGF2-forward | GTGGCATCGTTGAGGAGTG |
| IGF2-reverse | CACGTCCCTCTCGGACTTG |
| FOXM1-forward | GGAGCAGCGACAGGTTAAGG |
| FOXM1-reverse | GTTGATGGCGAATTGTATCATGG |
| CA9-forward | CTTTGCCAGAGTTGACGAGG |
| CA9-reverse | CGATTTCTTCCAAGCGAGAC |
| β-actin-forward | AGCACAGAGCCTCGCCTTT |
| β-actin-reverse | ATCATCATCCATGGTGAGCTGG |
| ETS1-forward | GCCAGTCATCTTTCAACAG |
| ETS1-reverse | CACGGTCCCGCACATAG |
| E-cadherin-forward | ACGCATTGCCACATACACTC |
| E-cadherin-reverse | GGTGTTCACATCATCGTCCG |
| miR-4521 | GCTAATTAAGTCCTGTGCTCAG |

**Table S5. Correlation between clinicopathological parameters and miR-4521 levels in 140 cases of GC tissues (cohort A, χ2 test).**

| **Viable** | **All cases** |  | **miR-4521** | | |
| --- | --- | --- | --- | --- | --- |
|  |  |  | **Low** | **High** | **P value** |
| **Age (years)**  < 60  ≥60 | 73  67 |  | 35 (47.9%)  35 (52.2%) | 38 (52.1%)  32 (47.8%) | 0.612 |
| **Gender**  Male  Female | 87  53 |  | 41 (47.1%)  29(54.7%) | 46 (52.9%)  24 (45.3%) | 0.384 |
| **Invasion depth**  T1/2  T3/4 | 52  88 |  | 20 (38.5%)  50 (56.8%) | 32 (61.5%)  38 (43.2%) | **0.036** |
| **TNM stage**  I/II  III/IV | 54  86 |  | 18 (33.3%)  52 (60.5%) | 36 (66.7%)  34 (39.5%) | **0.002** |
| **Lymph node metastasis**  N0/1  N2/3 | 56  84 |  | 16 (28.6%)  54 (64.3%) | 40 (71.4%)  30 (35.7%) | **<0.001** |
| **Distant metastasis**  Absent  Present | 123  17 |  | 56 (45.5%)  14 (82.4%) | 67 (54.5%)  3 (17.6%) | **0.004** |

**Table S6.** **Univariate and multivariate analyses of factors associated with overall survival in cohort B samples.**

| **Viable** | **Univariate analysis** | |  | **Multivariate analysis** | |
| --- | --- | --- | --- | --- | --- |
|  | **HR (95% CI)** | ***P* value** |  | **HR (95% CI)** | ***P* value** |
| Age (≥65 vs.<65) | 1.377 (0.803-2.363) | 0.245 |  | / | / |
| Gender (female vs. male) | 0.606 (0.310-1.183) | 0.142 |  | / | / |
| Tumor size (≥5cm vs. <5cm) | 1.894 (1.046-3.431) | 0.035 |  | 2.011 (1.103-3.665) | 0.023 |
| TNM stage (III/IV vs. I/II) | 1.223 (0.510-2.935) | 0.652 |  | / | / |
| Lymph node metastasis (present vs. absent) | 1.398 (0.545-3.590) | 0.486 |  | / | / |
| miR-4521 expression (high vs. low) | 0.567 (0.303-1.062) | 0.076 |  | 0.476 (0.270-0.839) | 0.010 |

HR: Hazard ratio; 95% CI: 95% confidence interval

**Table S7. Univariate and multivariate analyses of factors associated with overall survival in cohort C samples.**

| **Viable** | **Univariate analysis** | |  | **Multivariate analysis** | |
| --- | --- | --- | --- | --- | --- |
|  | **HR (95% CI)** | ***P* value** |  | **HR (95% CI)** | ***P* value** |
| Age (≥65 vs.<65) | 1.751 (1.048-2.925) | 0.032 |  | 1.794 (1.076-2.994) | 0.025 |
| Gender (female vs. male) | 0.751 (0.453-1.244) | 0.266 |  | / | / |
| Tumor size (≥5cm vs. <5cm) | 1.911 (1.111-3.288) | 0.019 |  | 1.889 (1.101-3.241) | 0.021 |
| TNM stage (III/IV vs. I/II) | 1.828 (0.824-4.058) | 0.138 |  | 2.140 (1.166-3.927) | 0.014 |
| Lymph node metastasis (present vs. absent) | 1.384 (0.549-3.485) | 0.491 |  | / | / |
| miR-4521 expression (high vs. low) | 0.546 (0.315-0.946) | 0.031 |  | 0.565 (0.329-0.968) | 0.038 |
