# Supplementary material for: Hypoxia downregulated miR-4521 suppresses gastric carcinoma progression through regulation of IGF2 and FOXM1
Source: Mol Cancer. 2021 Jan 6;20:9. doi: 10.1186/s12943-020-01295-2 (PMC7786912; doi:10.1186/s12943-020-01295-2)
Supplement: Supplementary file 1 — Additional file 1. [file 12943_2020_1295_MOESM1_ESM.zip › Table S8.docx]

**Table S8. Target gene prediction of miR-4521 in three databases（genes in at least two databases）**

| **Target gene** | **miRWalk** | **DIANA-TarBase** | **miRTarBase** | **Target gene** | **miRWalk** | **DIANA-TarBase** | **miRTarBase** |
| --- | --- | --- | --- | --- | --- | --- | --- |
| NUFIP2 | + | + | + | GSPT1 | + | + | - |
| URM1 | + | - | + | PTBP1 | + | + | - |
| CHRFAM7A | + | - | + | FOXJ3 | + | + | - |
| REPIN1 | + | - | + | SPTLC1 | + | + | - |
| IGF2 | + | - | + | BICD2 | + | + | - |
| MTMR12 | + | - | + | MED22 | + | + | - |
| FOSL2 | + | - | + | TPGS2 | + | + | - |
| BMPR1A | + | - | + | DYNLL2 | + | + | - |
| IREB2 | + | - | + | DCTN5 | + | + | - |
| CCND2 | + | - | + | BCL2L11 | + | + | - |
| UBE2G1 | + | - | + | SOX11 | + | + | - |
| ANKRD13A | + | - | + | ZNF680 | + | + | - |
| CELF1 | + | - | + | OCRL | + | + | - |
| TRIM35 | + | - | + | TNRC6B | + | + | - |
| KLHL42 | + | - | + | PBXIP1 | + | + | - |
| MYCBP | + | - | + | UHMK1 | + | + | - |
| ZFAND3 | + | + | - | PEX26 | + | + | - |
| MATR3 | + | + | - | PFKFB3 | + | + | - |
| E2F3 | + | + | - | ATF7IP | + | + | - |
| RAB30 | + | + | - | BICD1 | + | + | - |
| VAPA | + | + | - | LIFR | + | + | - |
| IGF2BP1 | + | + | - | MIER3 | + | + | - |
| ZNF652 | + | + | - | SCAMP1 | + | + | - |
| SZRD1 | + | + | - | FOXK1 | + | + | - |
| CMTM4 | + | + | - | CBX3 | + | + | - |
| PCYOX1 | + | + | - | TACC1 | + | + | - |
| EPB41L5 | + | + | - | MOB1B | + | + | - |
| HNRNPUL1 | + | + | - | CHST11 | + | + | - |
| CNIH4 | + | + | - | MLEC | + | + | - |
| CNST | + | + | - | SPRED2 | + | + | - |
| MDM2 | + | + | - | ZFP91 | + | + | - |
| ZNF580 | + | + | - | RALGAPB | + | + | - |
| PIK3R3 | + | + | - | MBD5 | + | + | - |
| POGK | + | + | - | KMT2C | + | + | - |
| IPCEF1 | + | + | - | CBX2 | + | + | - |
| PAPOLA | + | + | - | CADM1 | + | + | - |
| PHACTR2 | + | + | - | FOXM1 | + | + | - |
| PARPBP | + | + | - | GDAP2 | + | + | - |
| OAS2 | + | + | - | ARF6 | + | + | - |
| NAA15 | + | + | - | NIN | + | + | - |
| IPO7 | + | + | - | ADARB1 | + | + | - |
| LDHA | + | + | - | FBXW11 | + | + | - |
