# Supplementary material for: Hypoxia downregulated miR-4521 suppresses gastric carcinoma progression through regulation of IGF2 and FOXM1
Source: Mol Cancer. 2021 Jan 6;20:9. doi: 10.1186/s12943-020-01295-2 (PMC7786912; doi:10.1186/s12943-020-01295-2)
Supplement: Supplementary file 2 — Additional file 2. [file 12943_2020_1295_MOESM2_ESM.docx]

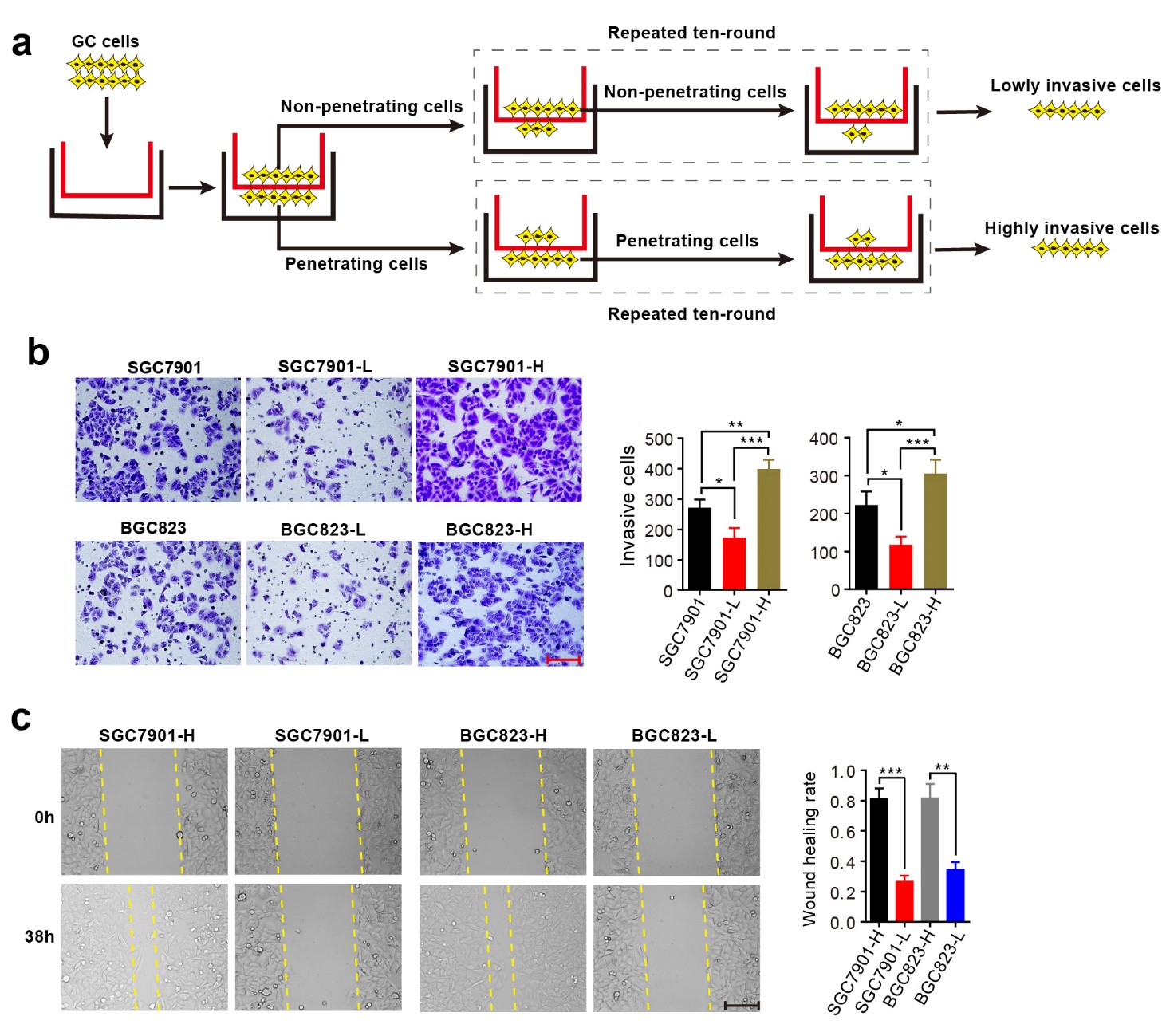


**Figure S1. Establishment of highly and lowly invasive GC cell sublines.** **a** General scheme of the establishment of highly invasive and lowly invasive cell sublines from SGC7901 and BGC823 cell lines. **b** Cell invasive abilities of parental, highly invasive and lowly invasive GC cell sublines. **c** Cell migratory abilities of highly and lowly invasive GC cell sublines. **b-c** Scale bars, 150 μm. Error bars indicate SD from 3 independent experiments. * *P* < 0.05; ** *P* < 0.01; ****P* < 0.001.


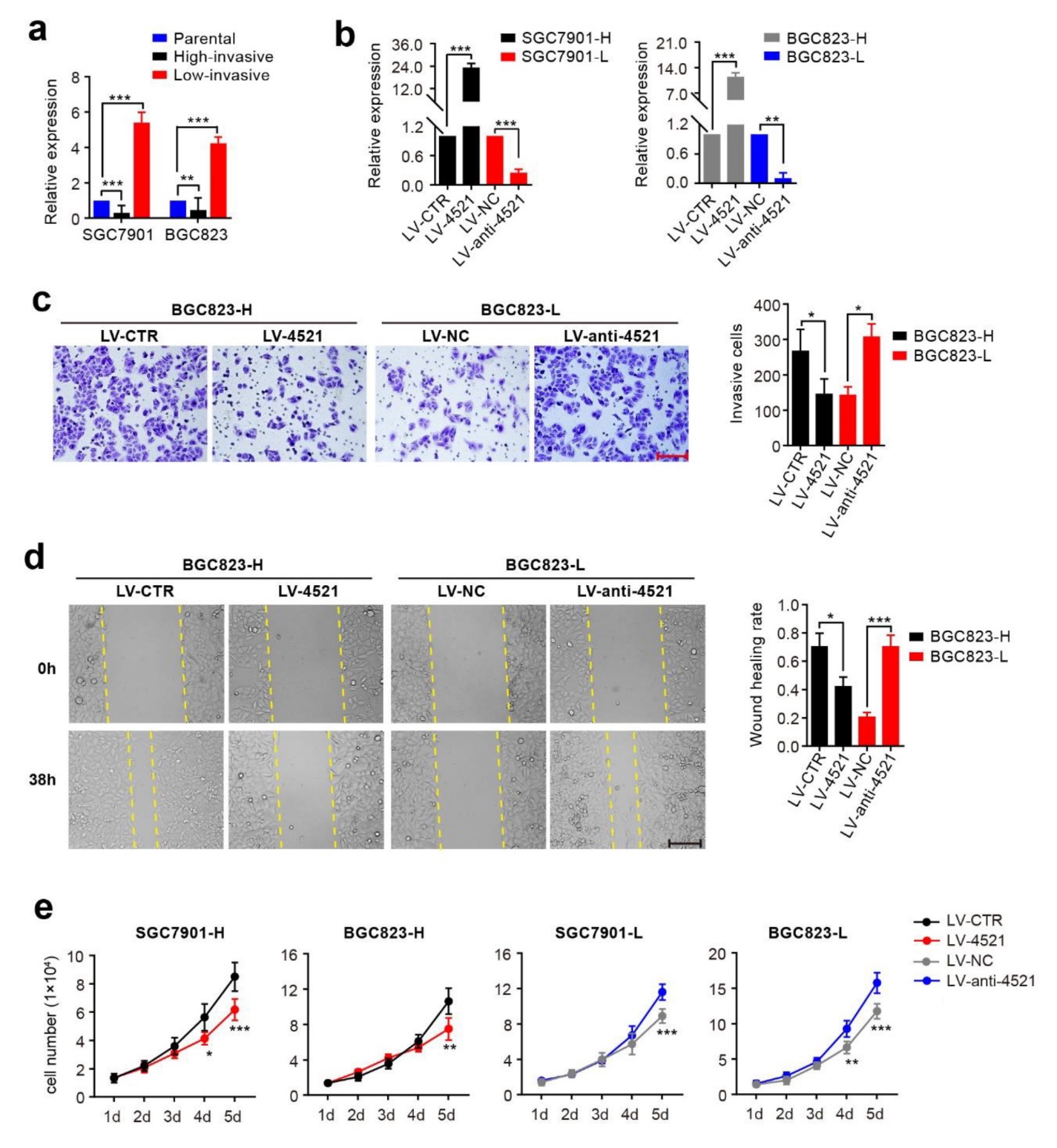


**Figure S2. miR-4521 inhibits GC cell invasion, migration and growth.** **a** qRT-PCR analysis of miR-4521 expression in parental, highly invasive and lowly invasive GC cell sublines. **b** qRT-PCR analysis was used to confirm the level of miR-4521 in cells with stable overexpression or knockdown of miR-4521. **c-d** Cell invasion and migration in BGC823-H cells stably expressing miR-4521, BGC823-L cells stably silencing miR-4521 and the respective control cells were examined by transwell and wound healing assays. Scale bars, 150 μm. **e** Cell proliferation assay in highly invasive cells stably expressing miR-4521, lowly invasive cells stably silencing miR-4521 and the control cells. **a-e** Error bars, SD from three independent experiments performed in triplicate. **P* < 0.05; ***P* < 0.01; ****P* < 0.001.


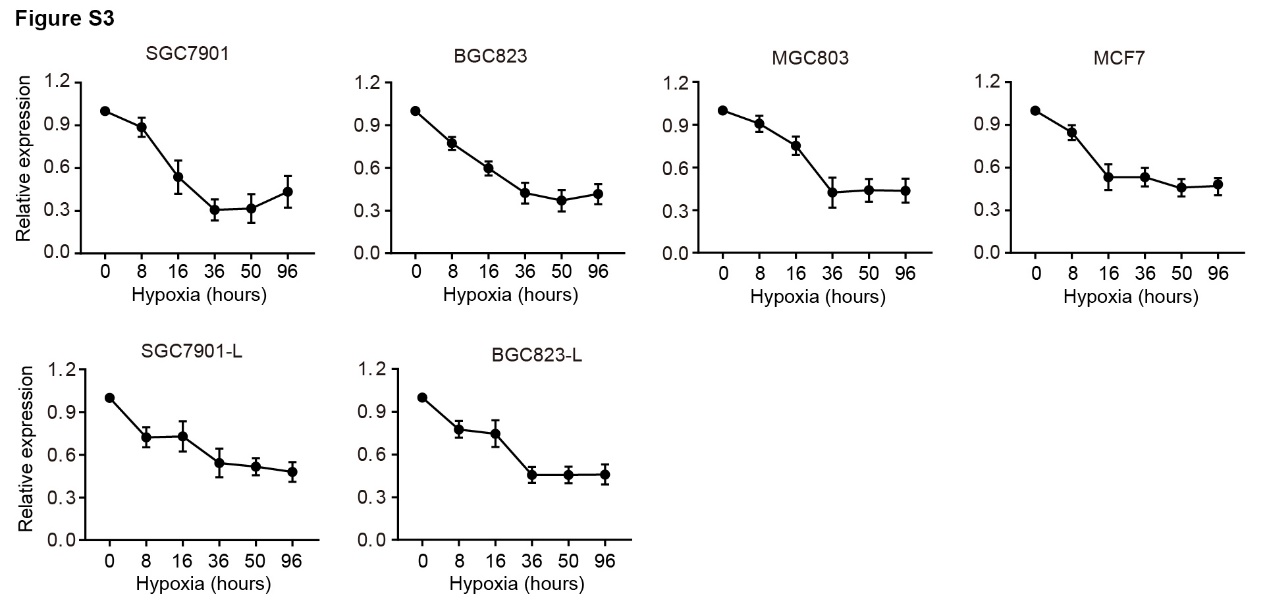


**Figure S3. miR-4521 expression levels under hypoxic conditions for multiple cancer cell lines at different time points.**

**
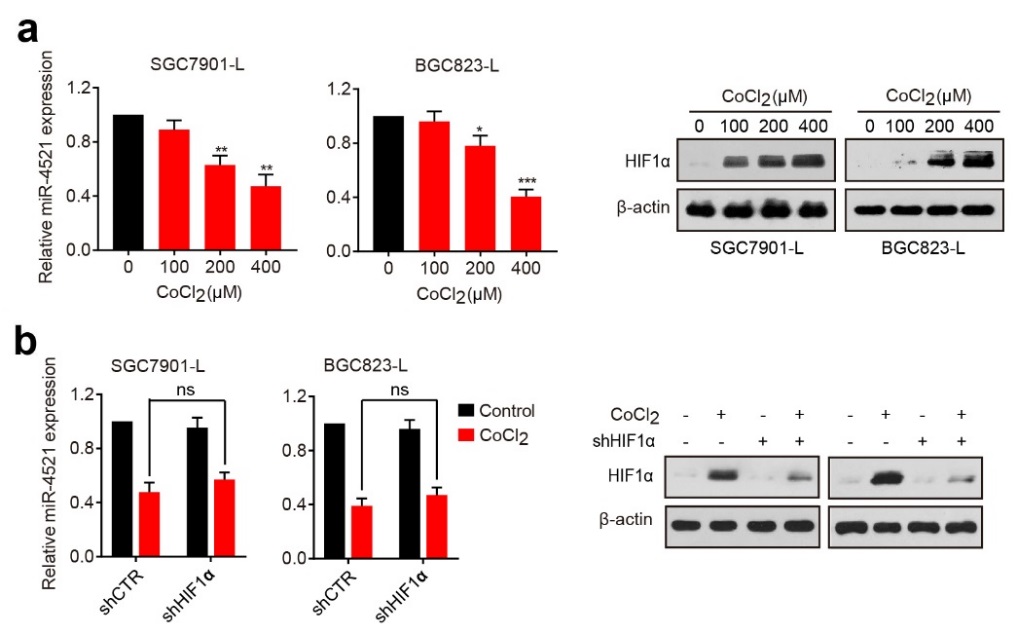
**

**Figure S4. CoCl_2_ treatment represses miR-4521 expression in a HIF1α-independent manner.** **a** qRT-PCR analysis of miR-4521 expression levels in SGC7901-L and BGC823-L cells incubated with different concentrations of CoCl_2_ for 16 h. Meanwhile, Western blotting was performed to detect HIF1α protein levels. **b** miR-4521 expression in HIF1α-silenced cells incubated with CoCl_2_ (400μM) for 16 h. Meanwhile, Western blotting analysis was performed to assess the inhibition efficiency. Error bars represent SD. **P* <0.05; ***P* < 0.01; ****P* <0.001; ns, not significant.


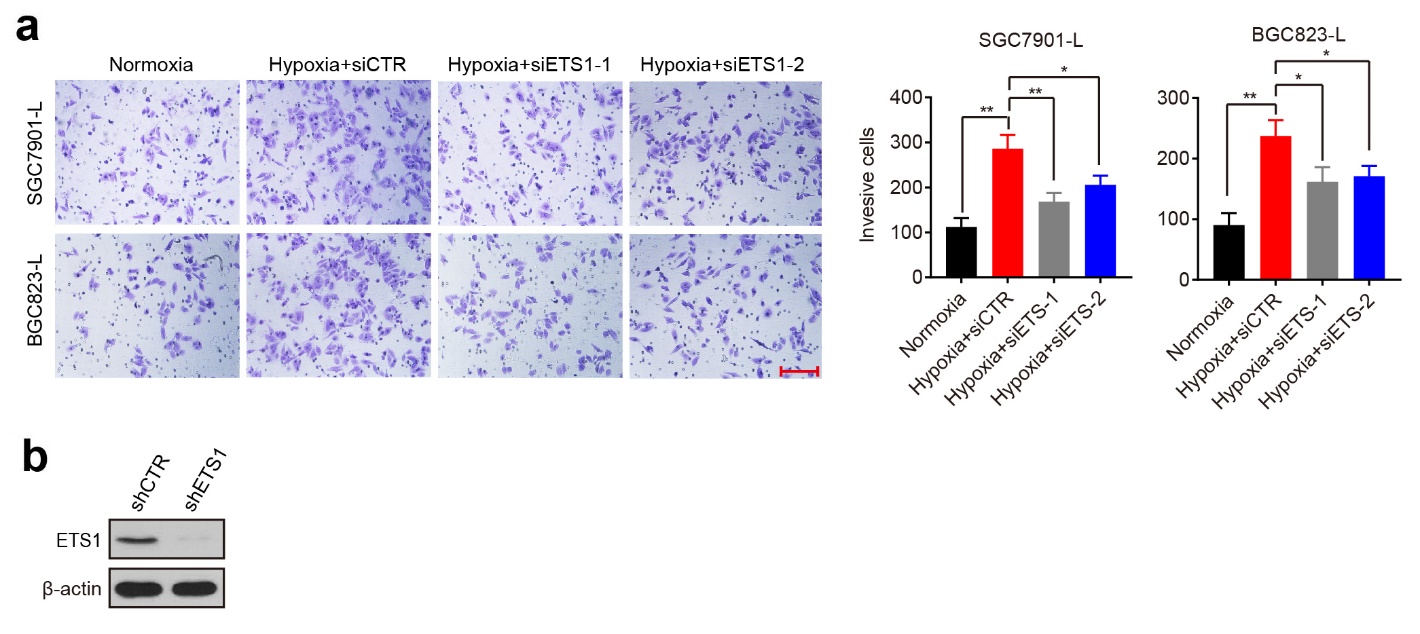


**Figure S5. Knockdown of ETS1 suppresses invasive ability of hypoxia-exposed cells.** **a** Cell invasion assays of SGC7901-L and BGC-L cells transfected with ETS1 siRNAs during hypoxia. Scale bar, 150 μm. Error bars indicate SD. **P* <0.05; ***P* < 0.01. **b** The inhibition efficiency was examined in SGC7901-H cells stably expressing ETS1 shRNA by Western blotting.

**
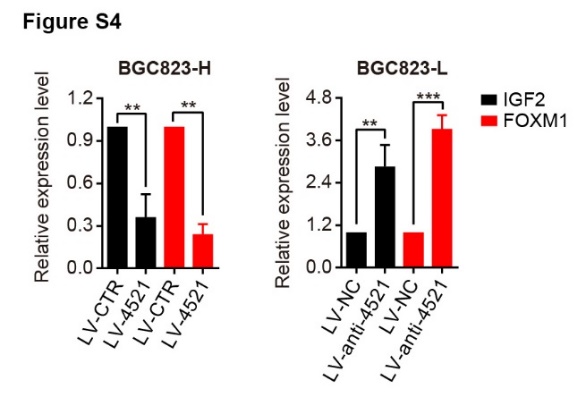
**

**Figure S6. miR-4521 downregulates IGF2 and FOXM1 mRNA levels.** Error bars denote SD. ***P* < 0.01; ****P* < 0.001.


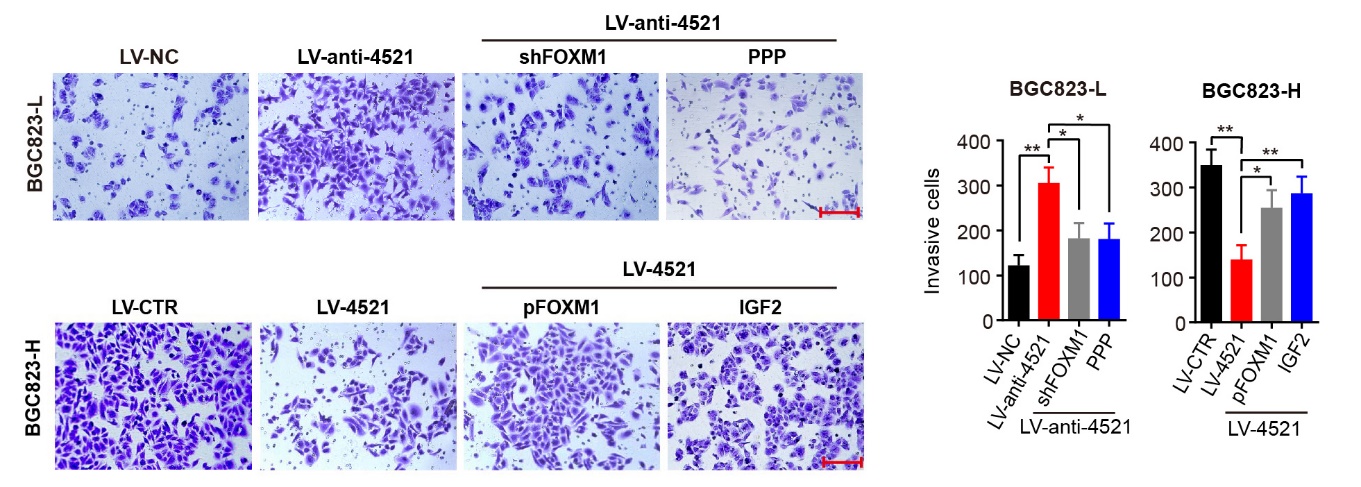


**Figure S7. IGF2 and FOXM1 offset the effect of miR-4521 on cell invasion.** miR-4521-silenced BGC823-L cells were transfected with FOXM1 siRNA or treated with the IGF1R inhibitor PPP (10 nM) for 48 hours, while BGC823-H cells overexpressing miR-4521 were transfected with a plasmid encoding FOXM1 or treated with 100 ng/ml IGF2 for 48 hours. The invasion ability of these cells was evaluated by transwell assay. Scale bars, 150 μm. Error bars denote SD. **P* < 0.05; ***P* < 0.01.


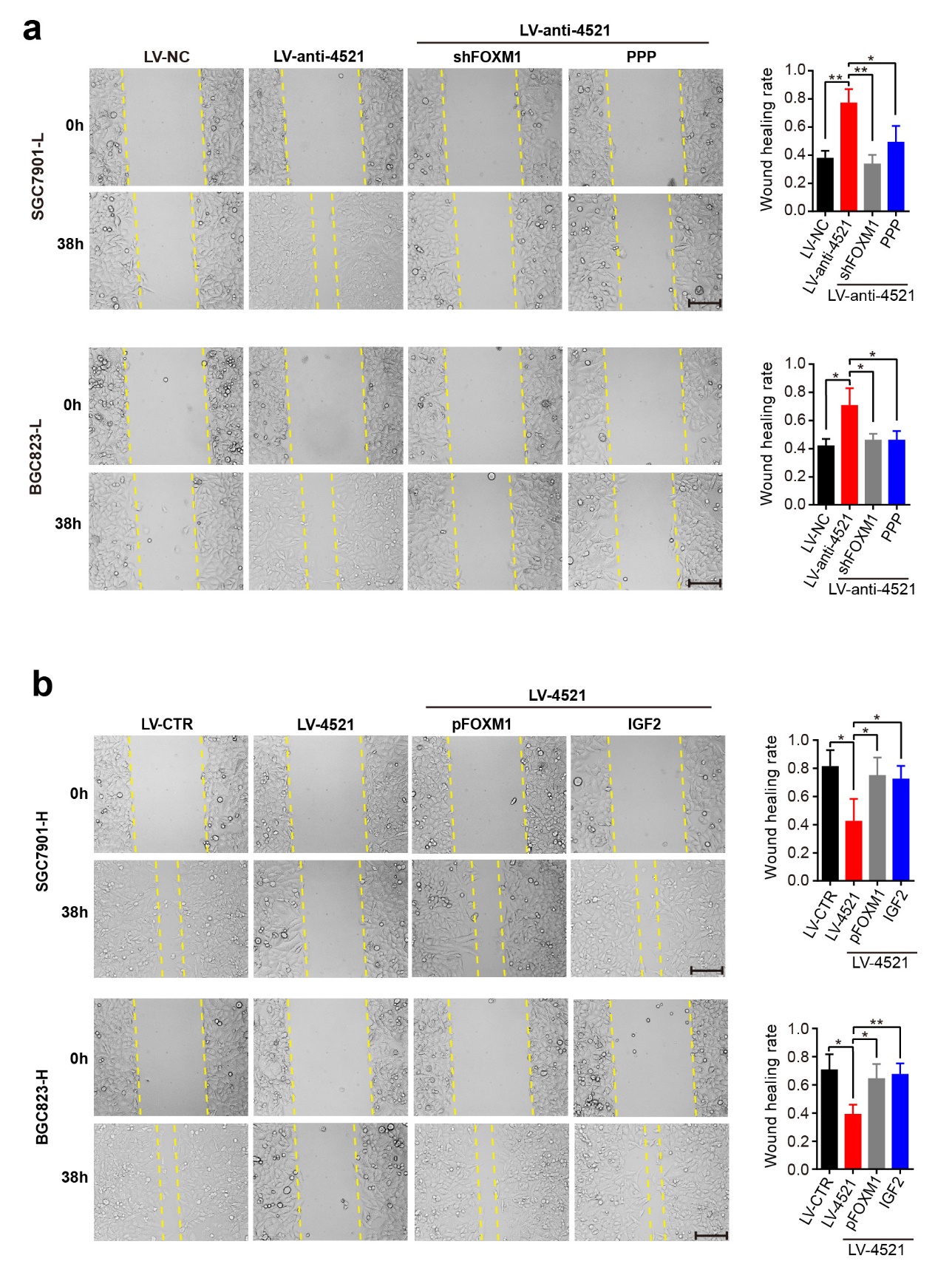


**Figure S8. IGF2 and FOXM1 abrogate the effect of miR-4521 on GC migration.** **a-b** miR-4521-silenced cells were transfected with FOXM1 shRNA or treated with the IGF1R inhibitor PPP (10 nM) for 38 hours **(a)**, while miR-4521-expressing cells were transfected with a plasmid encoding FOXM1 or treated with 100 ng/ml IGF2 for 38 hours **(b)**. The migratory ability of these cells was evaluated by transwell assay. Scale bars, 150 μm. Experiments were repeated three times, and error bars represent SD. **P* < 0.05; ***P* < 0.01.


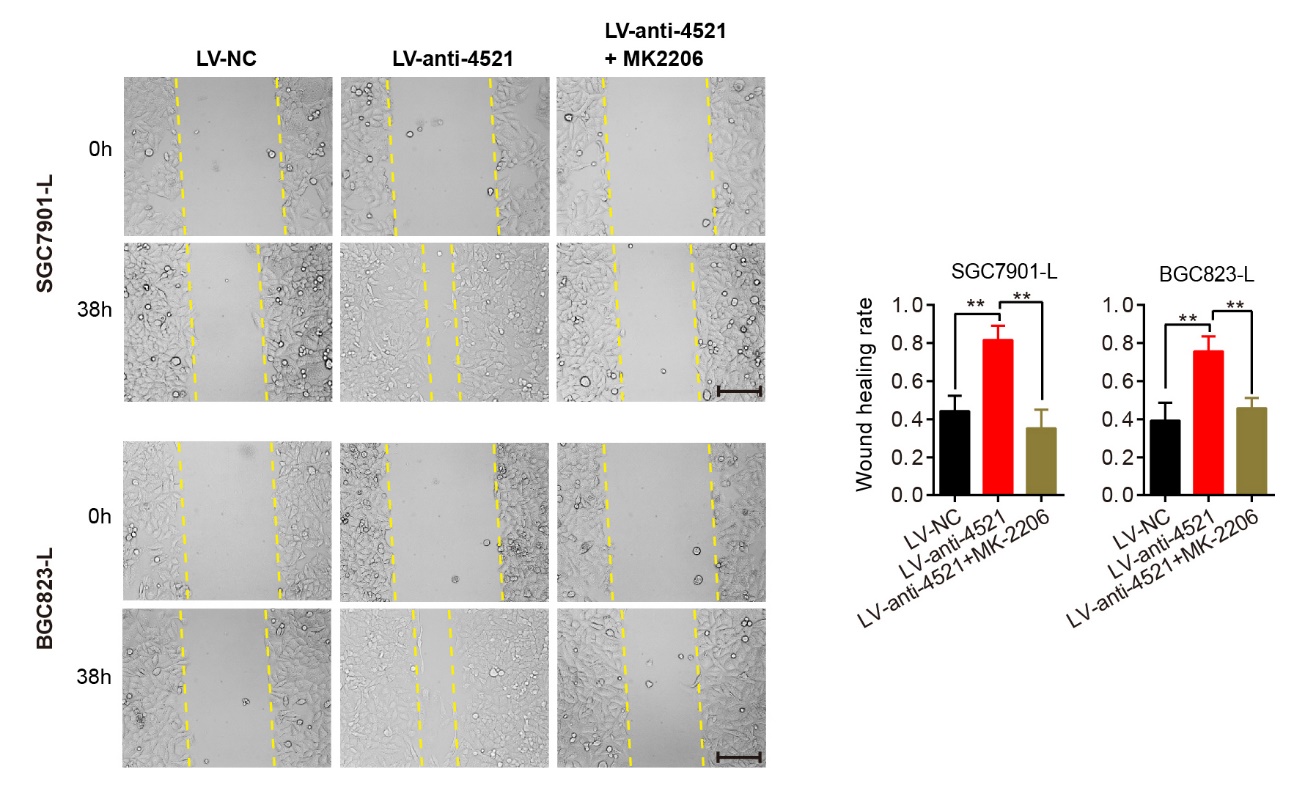


**Figure S9. Blocking the AKT pathway abrogates the effect of miR-4521 knockdown on cancer cell migration.** Cell migration was assessed in miR-4521-silenced cells treated with the AKT inhibitor MK2206. Scale bars, 150 μm. Error bars, SD from three independent experiments performed in triplicate. ***P* < 0.01.


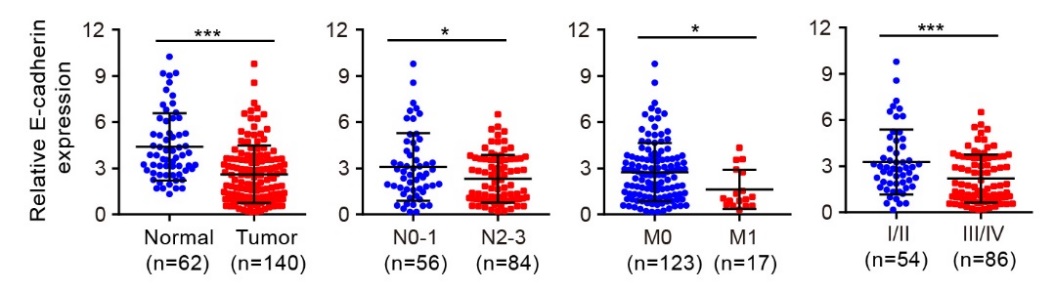


**Figure S10. E-cadherin mRNA levels** **in normal gastric tissues and GC tumors (cohort A).** Error bars represent SD. **P* < 0.05; ****P* < 0.001.
